# Supplementary material for: Biosynthesized gold nanoparticles that activate Toll-like receptors and elicit localized light-converting hyperthermia for pleiotropic tumor immunoregulation
Source: Nat Commun. 2023 Aug 24;14:5178. doi: 10.1038/s41467-023-40851-4 (PMC10449932; doi:10.1038/s41467-023-40851-4)
Supplement: Supplementary file 1 — Supplementary Information [file 41467_2023_40851_MOESM1_ESM.pdf]

Biosynthesized gold nanoparticles that activate Toll-like receptors and elicit localized light-converting hyperthermia for pleiotropic tumor immunoregulation

Hao Qin[1#], Yang Chen[1,2#], Zeming Wang[1,2], Nan Li[1,2], Qing Sun[1,2], Yixuan Lin[1,2], Wenyi Qiu[1,2], Yuting Qin[1], Long Chen[1], Hanqing Chen[3], Yiye Li[1], Jian Shi[1], Guangjun Nie[1,2,4✉] and Ruifang Zhao[1,4✉]

[1]CAS Key Laboratory for Biomedical Effects of Nanomaterials and Nanosafety, CAS Center of Excellence in Nanoscience, National Center for Nanoscience and Technology, Beijing 100190, P. R. China

[2]Center of Materials Science and Optoelectronics Engineering, University of Chinese Academy of Sciences, Beijing 100049, P. R. China

[3]Beijing Key Laboratory of Environmental Toxicology, Department of Toxicology and Sanitary Chemistry, School of Public Health, Capital Medical University, Beijing 100069, China

[4]GBA National Institute for Nanotechnology Innovation, Guangdong 510700, P. R. China

[#]These authors contributed equally: Hao Qin, Yang Chen.

Correspondence should address to G.N. or R.Z.

✉Email: niegj@nanoctr.cn (G.N.); zhaorf@nanoctr.cn (R.Z.)

Supplementary Table 1. Most abundant lipids in Ausome via lipidomics analysis

Lipids	Category	Abundance (Mean ± SD)	Ion modes
CL	Cardiolipin	0	positive
		33949.6 ± 25035.4	negative
PA	Glycerophospholipids	924438.0 ± 239417.5	positive
		19680687.1 ± 1651091.9	negative
PC	Glycerophospholipids	63397925.0 ± 82504378.0	positive
		8911900.0 ± 1666469.0	negative
PE	Glycerophospholipids	47449902.1 ± 24932578.7	positive
		165399063.2 ± 47839919.2	negative
PG	Glycerophospholipids	12911089.6 ± 1273895.0	positive
		15715490.3 ± 1111473.7	negative
PI	Glycerophospholipids	1207238.8 ± 1038186.6	positive
		2086701.6 ± 2038855.9	negative
PS	Glycerophospholipids	5134058.2 ± 2220920.9	positive
		8217363.7 ± 6612162.8	negative
DG	Glycerolipids	194451440.2 ± 1905920.5	positive
		31532179.4 ± 2336095.5	negative
MG	Glycerolipids	80800.9 ± 10785.6	positive
		4709099.6 ± 197270.9	negative
TG	Glycerolipids	9391543.5 ± 1323218.4	positive
		599742.4 ± 10357.4	negative
Cer	Sphingolipids	4291483.2 ± 2254619.8	positive
		5828375.5 ± 4629739.0	negative
GalCer	Sphingolipids	2093628.0 ± 2725372.5	positive
		3127737.2 ± 3762388.9	negative
SM	Sphingolipids	77521950.6 ± 109244708.5	positive
		1122312.3 ± 1167004.5	negative
Lipid A	Saccharolipids	3150958.7 ± 524711.6	positive
		0	negative

Supplementary Table 2. Selected membrane proteins identified in Ausome via proteomics analysis

Proteins	Gene symbol	Molecular weight (KDa)	Location	Unique peptides	Intensity
**Outer membrane protein C**	ompC	40.368	Outer membrane	3	690050000
**Outer membrane protein A**	ompA	37.2	Outer membrane	10	601030000
**Major outer membrane lipoprotein Lpp**	lpp	8.3234	Outer membrane	4	452420000
**Maltoporin**	lamB	49.912	Outer membrane	3	209220000
**Outer membrane protein assembly factor BamB**	bamB	41.887	Outer membrane	3	124860000
**Outer membrane protein assembly factor BamA**	bamA	90.552	Outer membrane	3	8266900
**Outer membrane lipoprotein RcsF**	rcsF	14.163	Outer membrane	3	55956000
**Long-chain fatty acid transport protein**	fadL	48.541	Outer membrane	2	29717000
**Osmotically-inducible protein Y**	osmY	21.073	Outer membrane-bounded periplasmic space	6	218940000
**Probable phospholipid-binding protein MlaC**	mlaC	23.962	Outer membrane-bounded periplasmic space	5	40995000
**Fructose-bisphosphate aldolase class 2**	fbaA	39.147	Plasma membrane	4	122990000
**Chaperone protein DnaK**	dnaK	69.114	Plasma membrane	4	57205000
**Multidrug resistance protein MdtE**	mdtE	41.19	Plasma membrane	3	37330000
**Chaperone protein HtpG**	htpG	71.422	Plasma membrane	3	20059000
**Elongation factor Tu2/Tu1**	tufB/A	43.313	Plasma membrane	8	355620000
**Protein-export membrane protein SecG**	secG	11.365	Plasma membrane	0	0
**Probable lipoprotein YiaD**	yiaD	22.197	Cell membrane	2	216920000
**D-methionine-binding lipoprotein MetQ**	metQ	29.431	Cell membrane	2	299210000

Supplementary Table 3. Hematological parameters estimations in rats treated with Ausome at the termination of study.

Parameters	Groups (Female)			Groups (Male)		
	Ctrl	LD	HD	Ctrl	LD	HD
WBC (× 10^9/L)	2.51 ± 1.02	2.76 ± 0.97	1.93 ± 0.79	1.41 ± 0.26	2.30 ± 0.67	1.97 ± 0.79
Neu %	12.97 ± 5.03	12.40 ± 2.40	11.23 ± 0.53	12.30 ± 1.28	16.87 ± 2.62	13.47 ± 4.00
Lym %	83.80 ± 7.17	85.20 ± 2.00	85.10 ± 1.28	83.97 ± 3.38	78.63 ± 2.26	80.73 ± 7.07
Mon %	2.37 ± 1.84	2.93 ± 0.78	2.90 ± 0.93	3.17 ± 2.17	4.13 ± 0.54	5.30 ± 4.38
Eos %	0.60 ± 0.43	0.10 ± 0.08	0.33 ± 0.21	0.23 ± 0.33	0.20 ± 0.08	0.13 ± 0.05
Bas %	0.27 ± 0.09	0.20 ± 0.08	0.43 ± 0.19	0.33 ± 0.12	0.17 ± 0.12	0.37 ± 0.25
RBC (× 10^{12}/L)	6.54 ± 0.30	6.56 ± 0.15	6.69 ± 0.22	6.81 ± 0.41	7.09 ± 0.13	6.56 ± 0.29
HGB (g/L)	133.00 ± 5.89	133.33 ± 0.47	136.00 ± 4.32	142.67 ± 6.55	144.33 ± 1.70	133.33 ± 4.64
HCT (%)	36.43 ± 1.86	36.87 ± 0.74	36.70 ± 0.65	39.33 ± 1.92	40.63 ± 0.91	36.43 ± 1.25
MCV (fL)	55.70 ± 0.86	56.23 ± 0.48	56.40 ± 0.73	57.80 ± 1.20	57.23 ± 0.19	55.57 ± 1.11
MCH (pg)	20.37 ± 0.25	20.37 ± 0.50	20.30 ± 0.00	21.00 ± 0.37	20.40 ± 0.24	20.33 ± 0.45
MCHC (g/L)	365.33 ± 2.62	362.00 ± 7.79	360.33 ± 4.50	362.67 ± 2.87	356.33 ± 5.44	365.67 ± 1.25
RDW-CV (%)	13.20 ± 0.08	13.70 ± 0.43	14.13 ± 1.05	14.30 ± 1.49	16.07 ± 1.46	14.03 ± 0.26
RDW-SD (fL)	29.13 ± 0.48	30.33 ± 1.24	31.50 ± 2.76	32.60 ± 2.84	36.40 ± 3.61	30.63 ± 1.03
PLT (× 10^9/L)	698.67 ± 79.37	717.33 ± 129.91	752.00 ± 22.55	759.33 ± 28.6395	789.67 ± 6.18	835.33 ± 29.68
MPV (fL)	5.67 ± 0.12	5.83 ± 0.36	5.63 ± 0.17	5.77 ± 0.37	6.10 ± 0.28	5.97 ± 0.12
PDW (fL)	6.17 ± 0.05	6.37 ± 0.62	6.03 ± 0.24	6.33 ± 0.58	6.83 ± 0.58	6.50 ± 0.28
PCT (%)	0.40 ± 0.04	0.42 ± 0.08	0.42 ± 0.00	0.44 ± 0.03	0.48 ± 0.02	0.50 ± 0.03

Data are presented as mean ± s.d. (n = 3). WBC, White blood cell counts; Neu, Neutrophils; Lym, Lymphocytes; Mon, Monocytes; Eos, Eosinophils; Bas, Basophils;

RBC, Red blood cell counts; HGB, Hemoglobin; HCT, Hematocrit; MCV, Mean corpuscular volume; MCH, Mean corpuscular hemoglobin; MCHC, Mean corpuscular hemoglobin concentration; RDW-CV, Red cell distribution width-coefficient variation; RDW-SD, Red cell distribution width-standard deviation; PLT, Platelets; MPV, Mean platelet volume; PDW, Platelet distribution width; PCT, Plateletcrit.

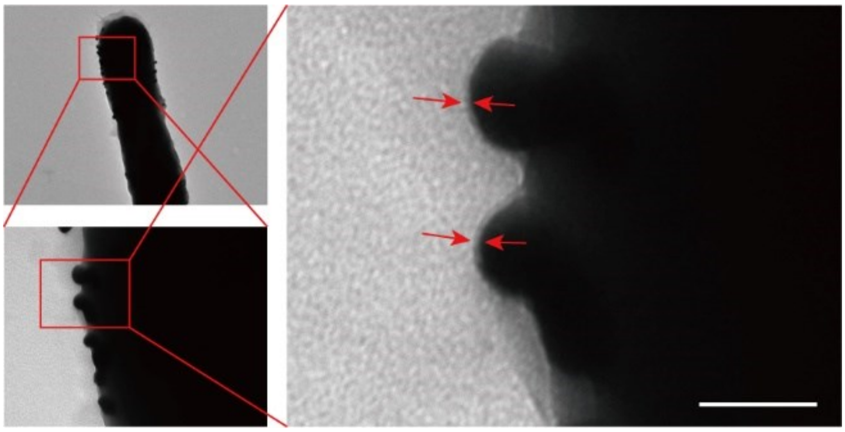

Supplementary Figure 1. TEM images of the bacterial compartment of gold nanoparticle generation. Representative TEM images of *E. coli* after incubation with $HAuCl_4$ for 48 h. The red arrows indicate the thin layer coating the nanoparticles at the margin of the bacterial cell. This experiment was repeated three times independently with similar results. Scale bar, 50 nm.

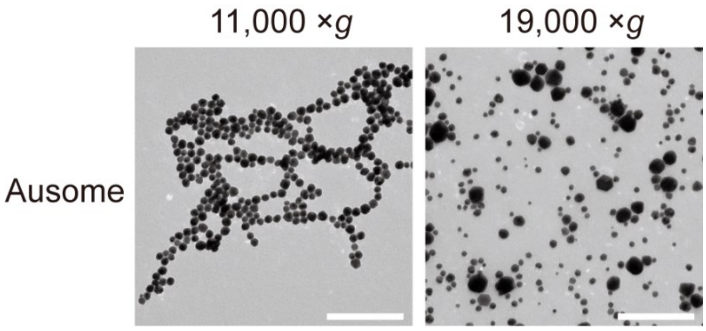

Supplementary Figure 2. Optimization of the centrifugation conditions for Ausome purification. TEM images of Ausome collected from crude extraction solutions through centrifugating at the indicated forces. This experiment was repeated three times independently with similar results. Scale bars, 200 nm.

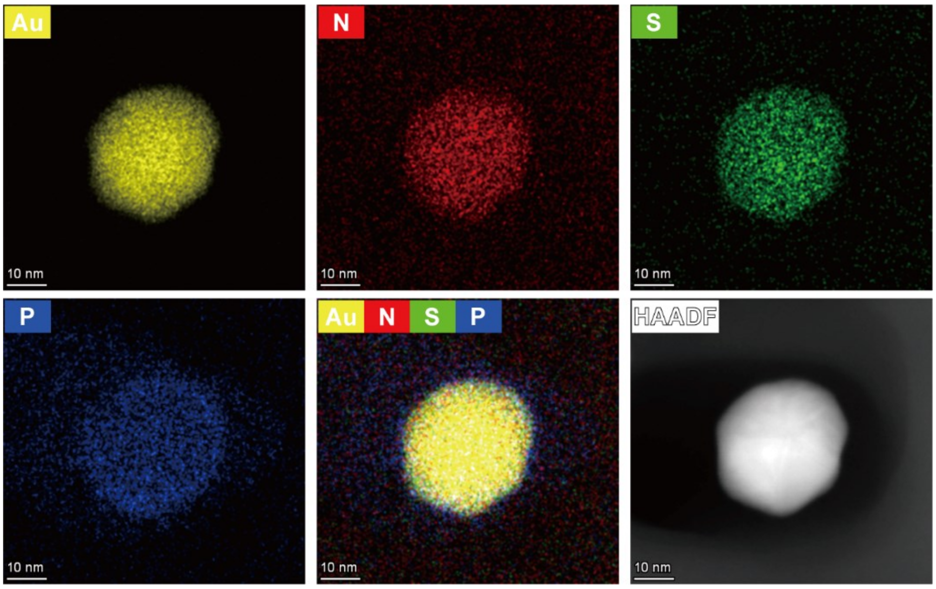

Supplementary Figure 3. Elemental composition and distribution of Ausome. EDS elemental mapping of the positive signals of Au and N, S, P, which indicate protein, phospholipid or nucleic acid components, and the corresponding high-angle annular dark-field (HAADF) STEM imaging of Ausome. This experiment was repeated three times independently with similar results. Scale bars, 10 nm.

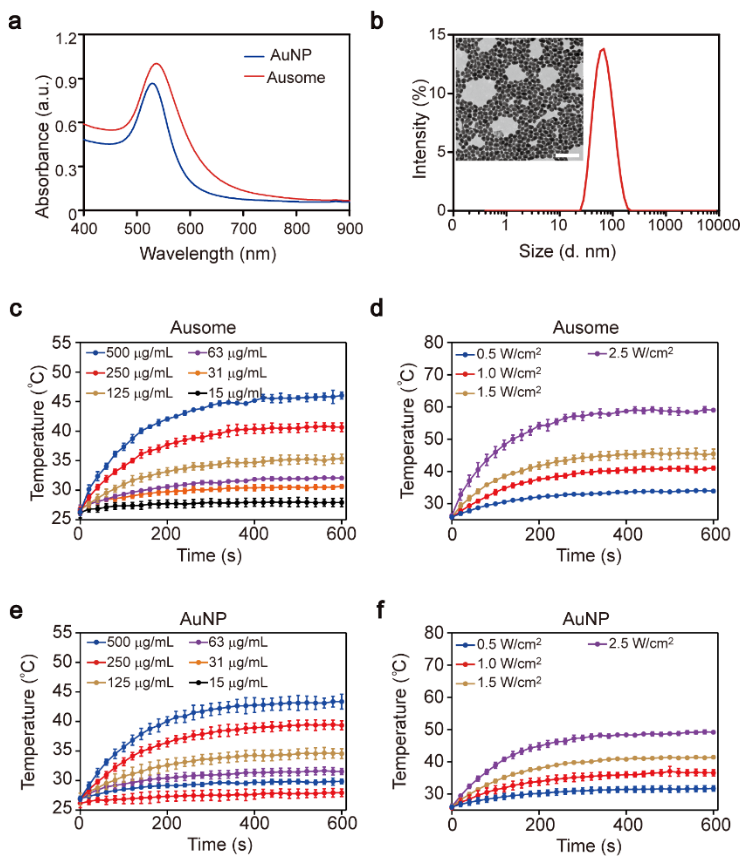

Supplementary Figure 4. Photothermal conversion of Ausome and chemically synthetic gold nanoparticles (AuNP). **a**, UV-vis absorption spectra of Ausome and AuNP. **b**, DLS detection of the size of AuNP in aqueous suspension. Inset: TEM image of AuNP, scale bar: 200 nm. **c,d**, Temperature changes of Ausome solution (n = 3 biologically independent experiments) under laser irradiation (660 nm) at the indicated concentrations (500, 250, 125, 63, 31 or 15 μg/mL, **c**) and power densities (0.5, 1.0, 1.5 or 2.5 W/cm^2, **d**). **e,f**, Temperature changes of AuNP (n = 3 biologically independent experiments) at the indicated concentrations (500, 250, 125, 63, 31 or 15 μg/mL, **e**) and exposed to laser irradiation with the indicated power density (0.5, 1.0, 1.5 or 2.5 W/cm^2), **f**). The numerical data in (**c-f**) are presented as the mean ± s.d. These experiments (**a, b**) were repeated three times independently with similar results. Source data are provided as a Source Data file.

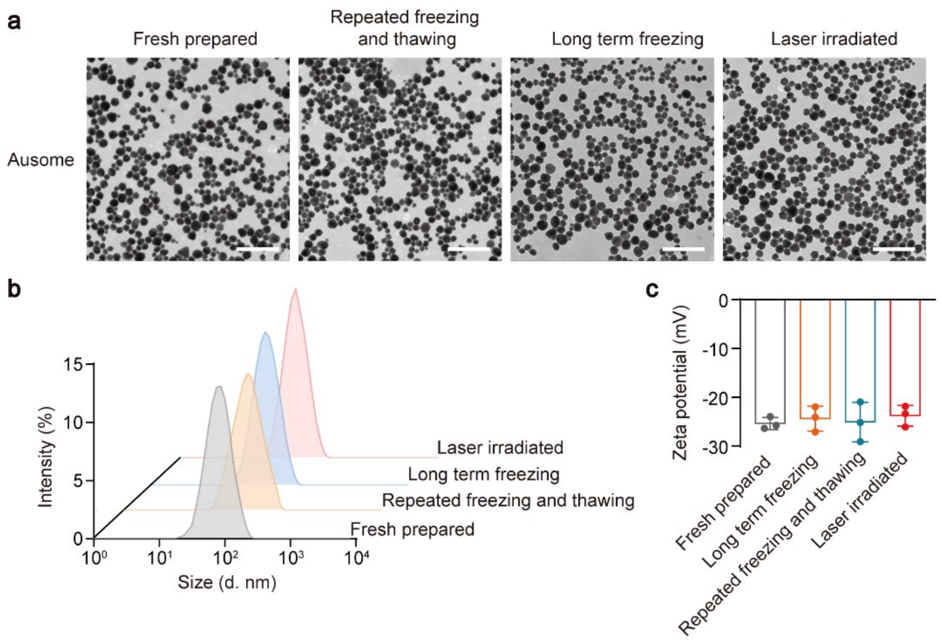

Supplementary Figure 5. Stability of Ausome under different storage conditions and laser irradiation. Representative TEM images (**a**), size distributions (**b**), and surface zeta potentials (n = 3 biologically independent experiments, **c**) of Ausome after repeated freezing and thawing (three cycles in one-week intervals, left), long time freezing (-80°C for 6 months) or laser irradiation (1.5 W/cm^2) for 30 min. Scale bars, 200 nm. The numerical data in (**c**) are presented as the mean ± s.d. Source data are provided as a Source Data file.

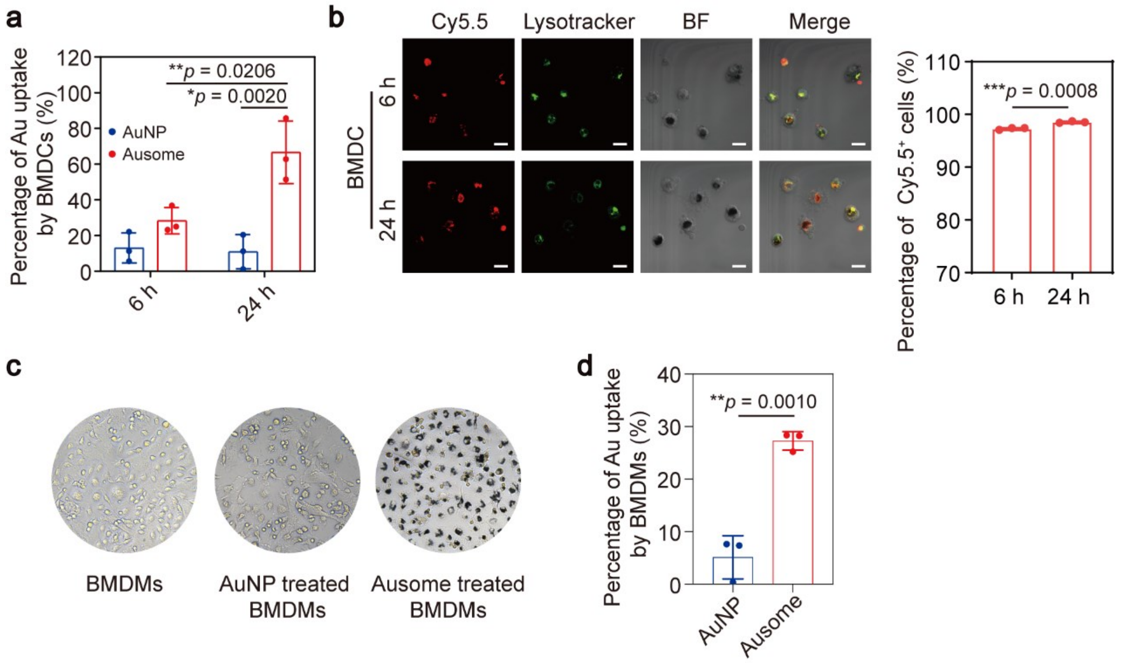

Supplementary Figure 6. Recognition and internalization of Ausome by innate immune cells. **a**, ICP-MS-quantified percentage of intracellular Au after incubation of BMDCs with Ausome for 6 or 24 h (n = 3 biological independent samples). **b**, Representative confocal microscopy images and flow cytometry analysis (n = 3 biological independent samples) of BMDCs treated with Cy5.5-labeled Ausome for 6 or 24 h (red: Cy5.5-labeled Ausome, green: Lysotracker). Scale bars, 20 µm. **c**, Bright field images of BMDMs after incubating with chemically synthesized AuNP or Ausome for 12 h. **d**, ICP-MS-quantified percentage of intracellular Au after incubation of BMDMs with Ausome for 12 h (n = 3 biological independent samples). The numerical data in (**a, b, d**) are presented as the mean ± s.d. *$p < 0.05$, **$p < 0.01$, **$p < 0.001$; significant differences were analyzed by one-way ANOVA followed by the Bonferroni multiple comparison test (**a)** or two-tailed unpaired *t*-test (**b, d**). This experiment (**c**) was repeated three times independently with similar results. Source data are provided as a Source Data file.

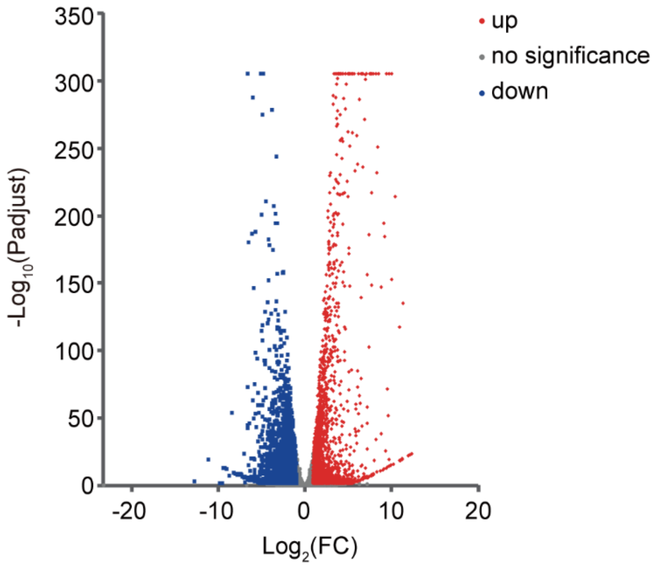

Supplementary Figure 7. Ausome-triggered differential gene expression in BMDCs. Comparative transcriptome analysis of BMDCs with or without Ausome treatment. The volcano plot represents the differentially expressed genes present in BMDCs treated for 12 h with Ausome (100 µg Ausome for 10^6 cells; n = 3 biologically independent samples), when compared with untreated BMDCs. The significantly upregulated gene points are presented in red, while the significantly downregulated genes are in blue. Differential expression gene data under the significance threshold ($p < 0.05$, foldchange > 2) are shown as grey dots. The statistical significances were determined by a two-tailed t-test, with the Benjamini-Hochberg method to correct *P*-values. Results were considered with significant differences at *P*-values < 0.05. Source data are provided as a Source Data file.

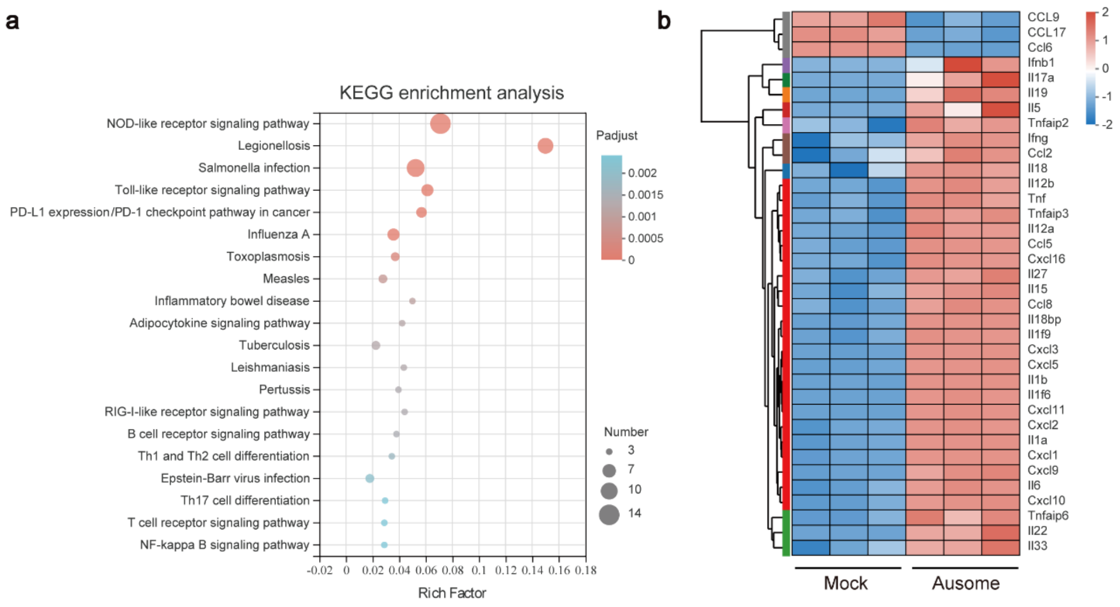

Supplementary Figure 8. Ausome-triggered immune responses in BMDCs. Transcriptome analysis of BMDCs with or without Ausome treatment (100 µg Ausome per 10^6 cells, n = 3 biologically independent samples). **a**, Bubble plot of KEGG pathway enrichment analysis of the 20 most affected signal pathways of the differentially expressed genes. **b**, Heat map presenting cluster analysis of the differential expression of cytokines and chemokines between Ausome-treated and untreated BMDCs. The statistical significances were determined by a two-tailed t-test, with the Benjamini-Hochberg method to correct *P*-values. Results were considered with significant differences at *P*-values < 0.05. Source data are provided as a Source Data file.

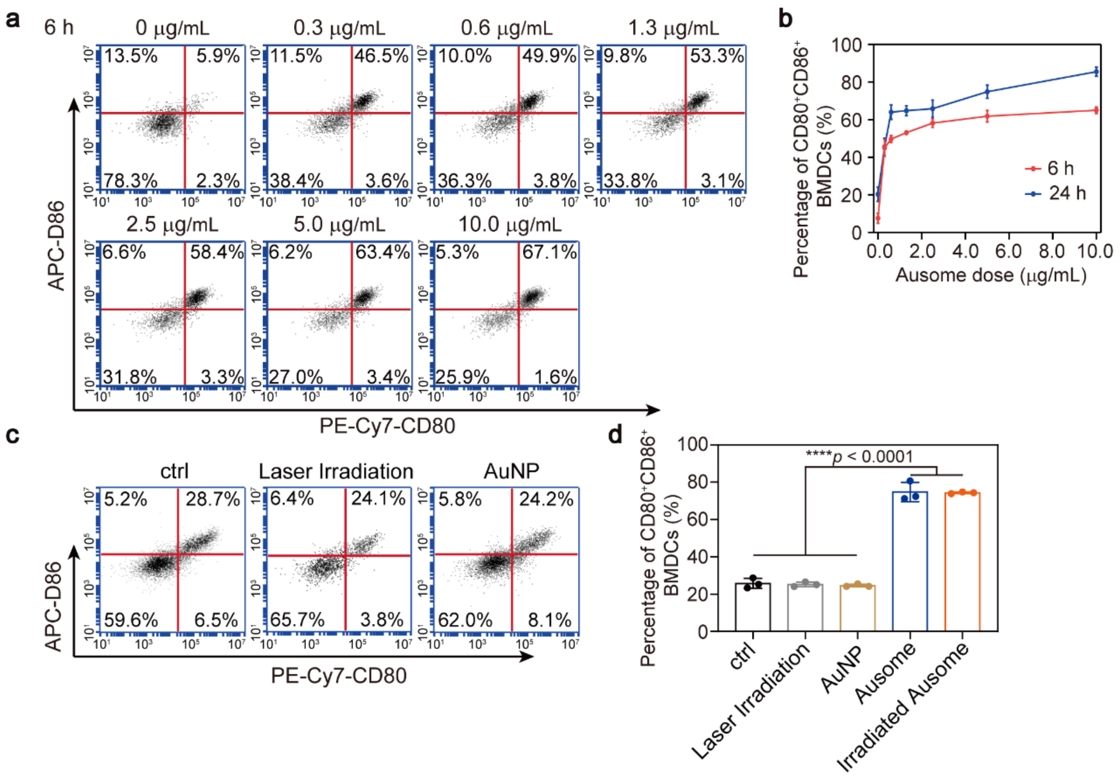

Supplementary Figure 9. Ausome-induced DC maturation. **a**, Representative flow cytometry dot plots of CD80$^+$CD86$^+$ BMDCs (2 ×10^5 cells per sample) after treatment with the indicated doses of Ausome for 6 h. **b**, Statistics chart of CD80$^+$CD86$^+$ BMDCs after Ausome treatment at the indicated doses and incubation times (n = 3 biological independent samples). **c**, Representative flow cytometry dot plots of BMDCs (2 × 10^5 cells per sample) stained with fluorescence-labeled antibodies against CD80 and CD86, after treating with 30 min laser irradiation or AuNP. **d**, Statistic analysis of CD80$^+$CD86$^+$ BMDCs after treating with 30 min laser irradiation, AuNP (2.5 μg/mL), Ausome (μg /mL) or irradiated Ausome (660 nm, 1.5 W/cm^2 laser irradiated for 30 min before incubating with BMDCs) for 24 h (n = 3 biological independent samples). The data are presented as the mean ± s.d. ****p < 0.0001; significant differences were analyzed by one-way ANOVA followed by the Bonferroni multiple comparison test (**d**). Source data are provided as a Source Data file.

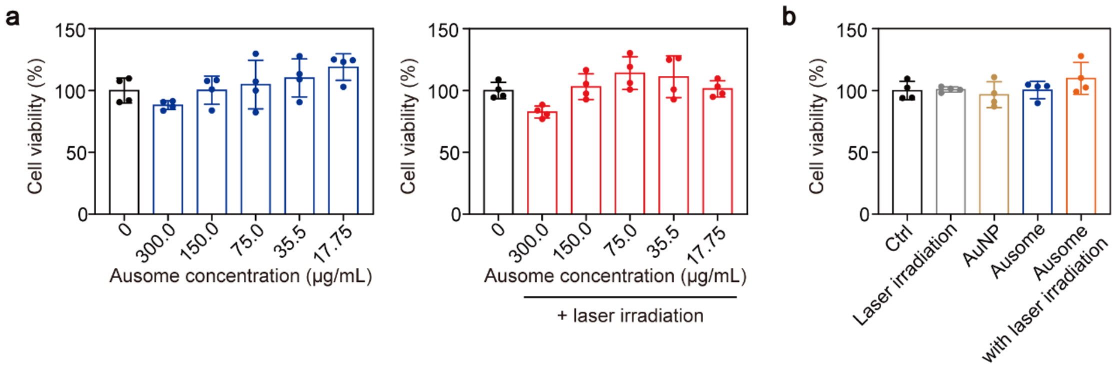

Supplementary Figure 10. Cytotoxicity of Ausome on tumor cells. **a**, 4T1 breast cancer cells were treated with Ausome at indicated doses (left histogram) or with Ausome and additional 30 min laser irradiation (right histogram). 24 h post these treatments, the cell viabilities were analyzed using Cell-Counting-Kit-8 assay (n = 4 biological independent samples). **b**, Viabilities of 4T1 cells after treating with 30 min laser irradiation, chemical synthetic AuNP (150 µg/mL), Ausome (150 µg/mL) or irradiated Ausome (660 nm, 1.5 W/cm^2 laser irradiated for 30 min before incubating with 4T1 cells) for 24 h (n = 4 biological independent samples). The data are presented as the mean ± s.d. Source data are provided as a Source Data file.

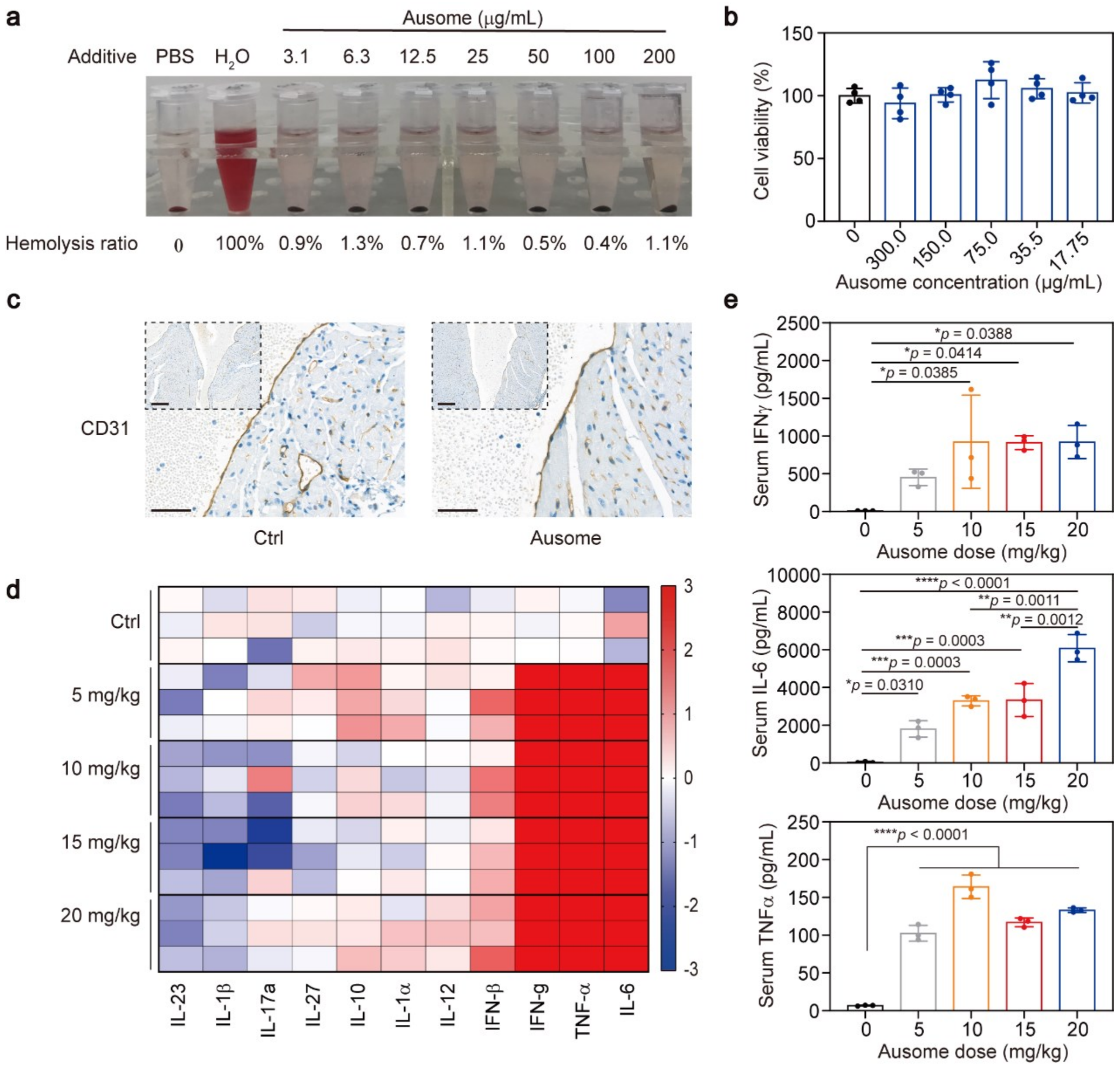

Supplementary Figure 11. Assessment of acute toxicity of Ausome. **a**, Representative images of the effects of Ausome on hemolysis (1 mL whole blood) at the indicated doses. Distilled water was used as a positive control. **b**, Cytotoxicity analysis of Ausome on vascular endothelial cells by treating human umbilical vein endothelial cells (HUVECs) with Ausome at different concentrations for 24 h, and cell viabilities were detected using Cell-Counting-Kit-8 assay (n = 4 biological independent samples). **c**, Heart sections from mice treated with high-dose Ausome (270 μg per mouse), which were then immune-stained with an antibody against CD31 to label vascular endothelial cells. Inserted boxes indicated high coverage. Scale bars, 50 μm and 200 μm (inserted images). **d,** Variations in cytokine levels in the serum from Balb/c mice at 12 h post intravenous injection of 0, 5, 10, 15 or 20 mg/kg Ausome, and the fold increase or decrease (3-fold or greater) when compared with the control group are

presented in a heat map (n = 3 mice). **e,** Serum concentrations of highly variable cytokines, including IFNγ, IL-6 and TNFα (n = 3 mice). The numerical data in **(b,e)** is shown as the mean ± s.d. *$p < 0.05$, **$p < 0.01$, ***$p < 0.001$, ****$p < 0.0001$; significant differences were analyzed by one-way ANOVA followed by the Bonferroni multiple comparison test. This experiment (**c**) was repeated three times independently with similar results. Source data are provided as a Source Data file.

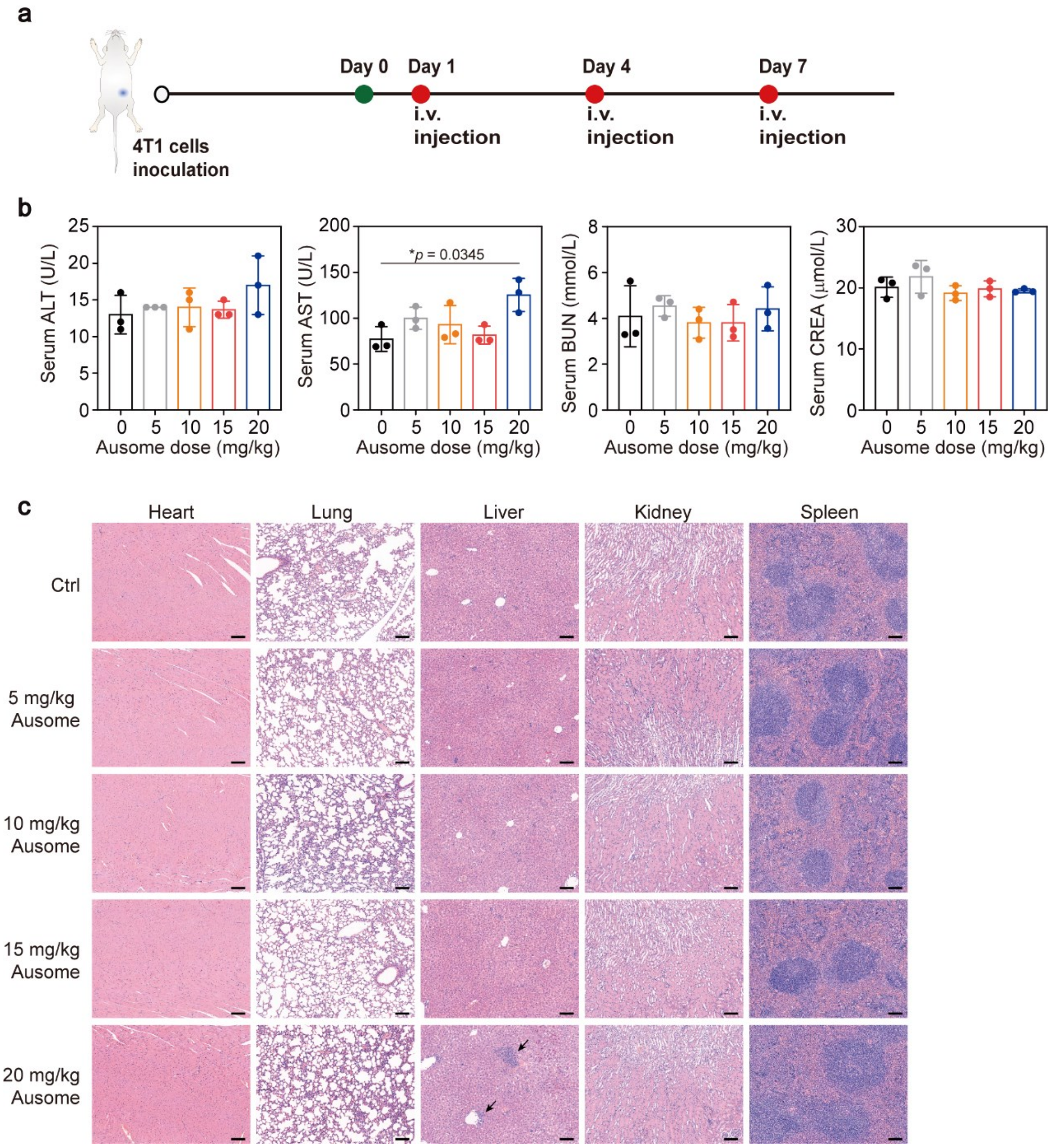

Supplementary Figure 12. *In vivo* long-term safety of Ausome. **a**, Schematic diagram of the Ausome treatment procedure. Balb/c mice were inoculated *in situ* with 4T1 tumor cells, followed by intravenous injections of Ausome on days 1, 4 and 7 at 0, 5, 10, 15 or 20 mg/kg, when tumors reached 50-100 mm^3 (day 0). **b,** Blood biochemistry analysis of alanine aminotransferase (ALT), aspartate aminotransferase (AST), blood urea nitrogen (BUN) and creatinine (CREA) in serum (day 16, n = 3 mice). **c,** Representative images of H&E stained organ sections from Ausome-treated mice (day 16). Black arrows indicate the infiltrating lymphocytes. Scale bars, 100 μm. The Numerical data in (**b**) are presented as the mean ± s.d. *$p < 0.05$; significant differences were analyzed by one-way ANOVA followed by the Bonferroni multiple comparison

test. This experiment (**c**) was repeated three times independently with similar results. Source data are provided as a Source Data file.

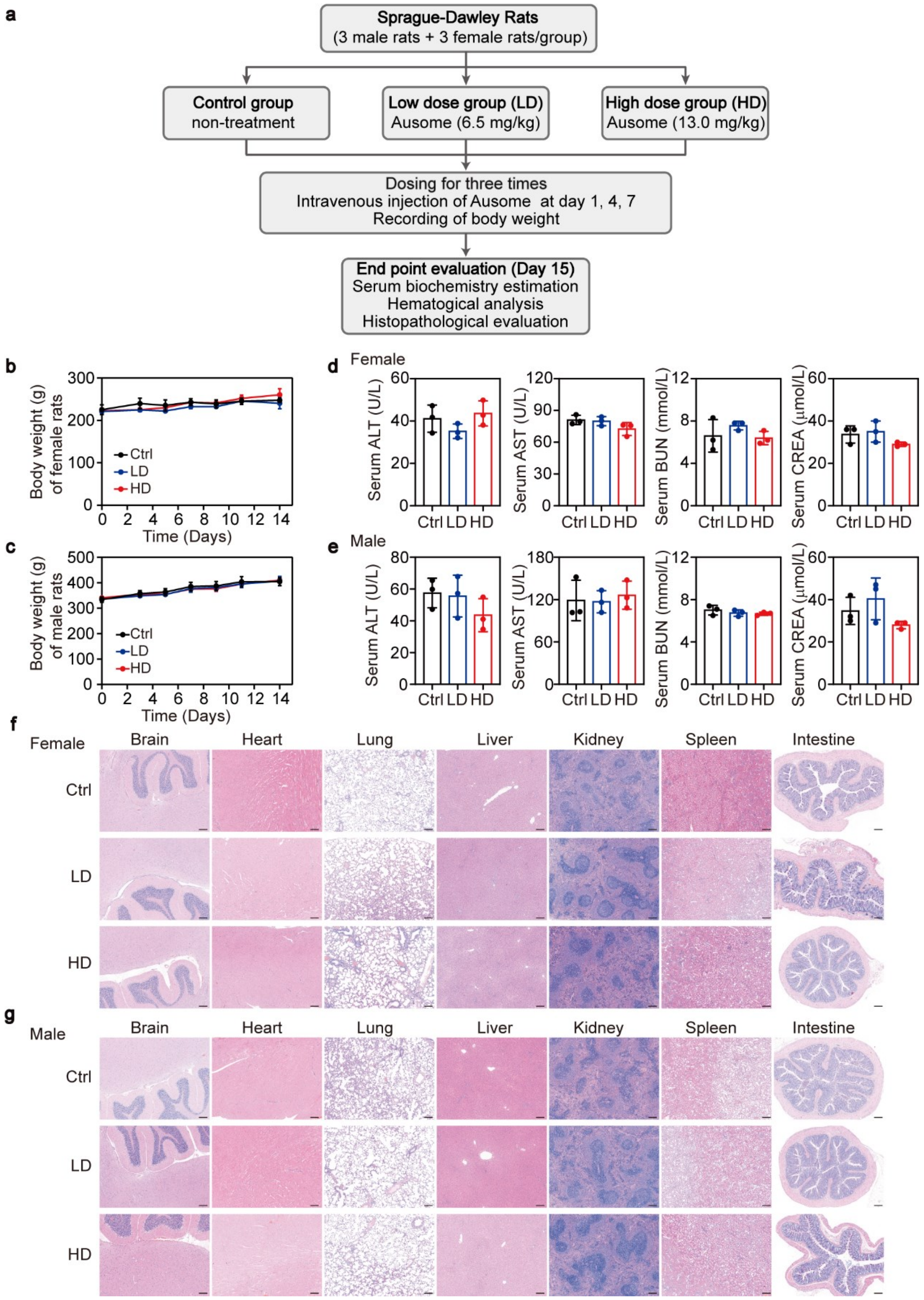

Supplementary Figure 13. Evaluation of Ausome biosafety profile in rats. **a**, Schematic illustration of experimental design of safety evaluation of Ausome in rats. Sprague-Dawley rats were divided into 3 groups, including non-treatment control group (Ctrl), low-dose treatment group (6.5 mg/kg Ausome, LD) and high-dose treatment

group (13 mg/kg Ausome, HD). And each group contained 3 female and 3 male rats. Ausome was intravenously injected for three times on days 1, 4 and 7. **b,c,** Body weights change of female rats (**b**) and male rats (**c**) throughout the study (n = 3 mice). **d,e,** Blood biochemistry analysis of ALT, AST, BUN and CREA in serum of female rats (**d**) and male rats (**e**) on day 15 (n = 3 mice). **f,g,** Representative images of H&E stained organs sections isolated from female rats (**f**) and male rats (**g**) on day 16. Scale bars, 200 μm. The numerical data in (**b-e**) are presented as the mean ± s.d. These experiments (**f, g**) were repeated three times independently with similar results. Source data are provided as a Source Data file.

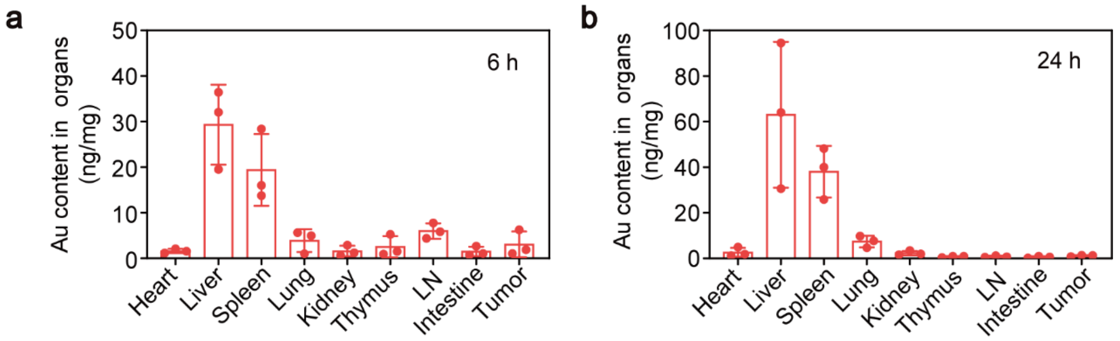

Supplementary Figure 14. Biodistribution of intravenously injected Ausome. **a**,**b**, ICP-MS detection of the Au content in the major organs from Balb/c mice at 6 h (**a**) or 24 h (**b**) post injection of 15 mg/kg Ausome (n = 3 mice). The data are presented as the mean ± s.d. Source data are provided as a Source Data file.

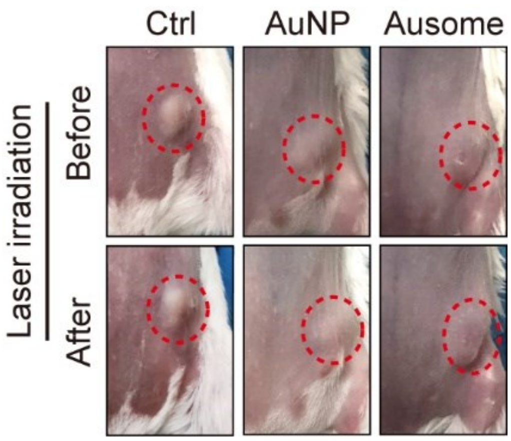

Supplementary Figure 15. Negligible effect of Ausome-mediated local hyperthermia elicited in tumor tissue. Representative photographs of Ausome-treated (15 mg/kg) Balb/c mice before (upper panel) and after (lower panel) laser irradiation (660 nm, 1.2 W/cm^2) for 30 min. This experiment was repeated three times independently with similar results. Dashed red circles indicate the tumor zone.

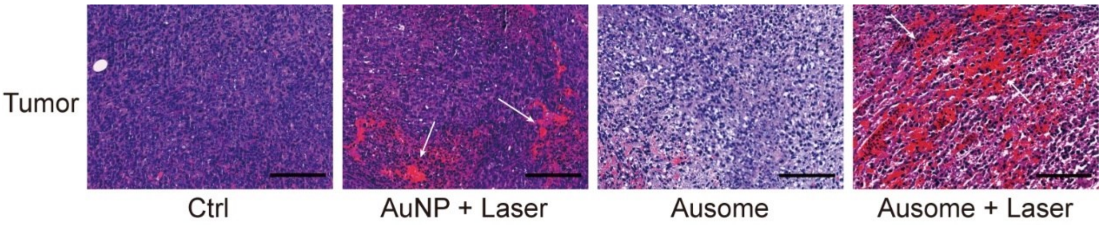

Supplementary Figure 16. Local hyperthermia-increased blood perfusion in tumors. Representative images of H&E stained tumor sections from mice treated with Ausome, AuNP (15 mg/kg) or Ausome (15 mg/kg) followed by laser irradiation (660 nm, 1.2 W/cm^2) for 30 min. The white arrows indicate perfused blood in the tumor tissue. Scale bars, 50 µm. This experiment was repeated three times independently with similar results.

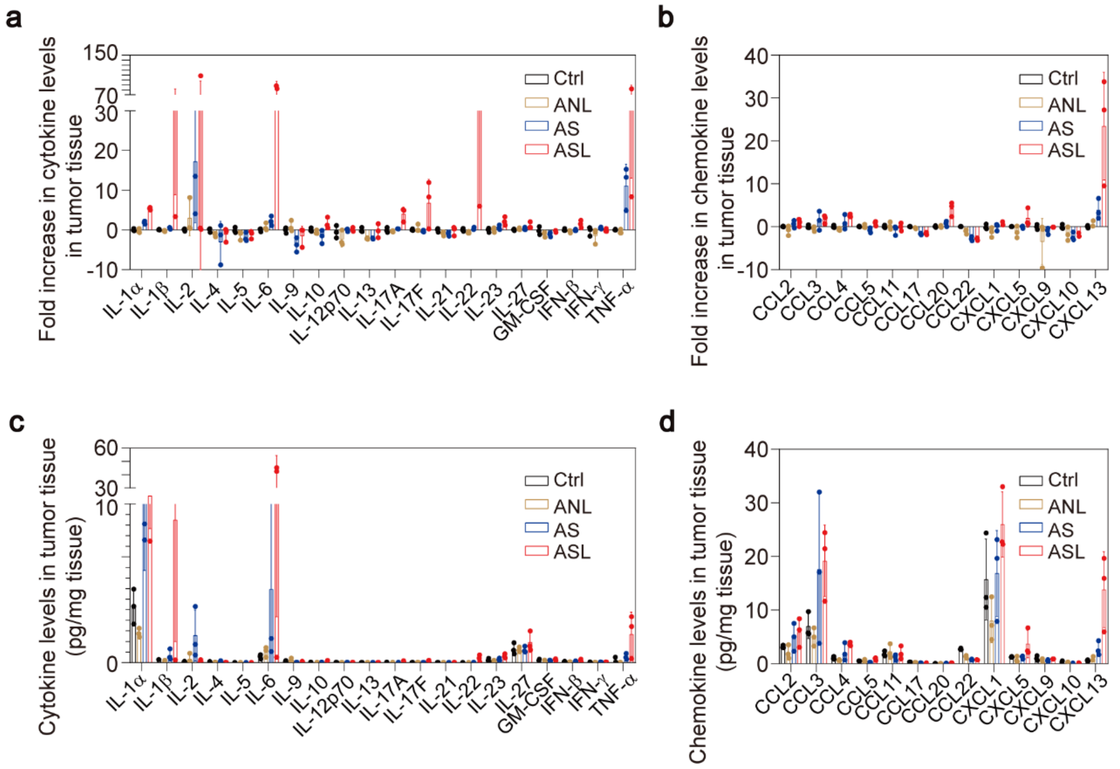

Supplementary Figure 17. Cytokine and chemokine release in the tumor area after Ausome-mediated, multi-layered modulation. Mice bearing 4T1 tumor were intravenously injected Ausome or AuNP (15 mg/kg) on day 1, followed by exposure to laser (660 nm, 1.2 W/cm^2) for 30 min at 6 h post injection. The procedure was repeated on day 7. 6 h after the second laser irradiation, the tumors were isolated, followed by measurement of the intratumoral cytokine and chemokine levels. AS group, Ausome injection without laser irradiation. n = 3 mice. **a,b**, Upregulated or downregulated cytokines (**a**) and chemokines (**b**), compared to the untreated control group. **c,d**, Concentration of cytokines (**c**) and chemokines (**d**) in the tumor tissue. The data are shown as the mean ± s.d. Source data are provided as a Source Data file.

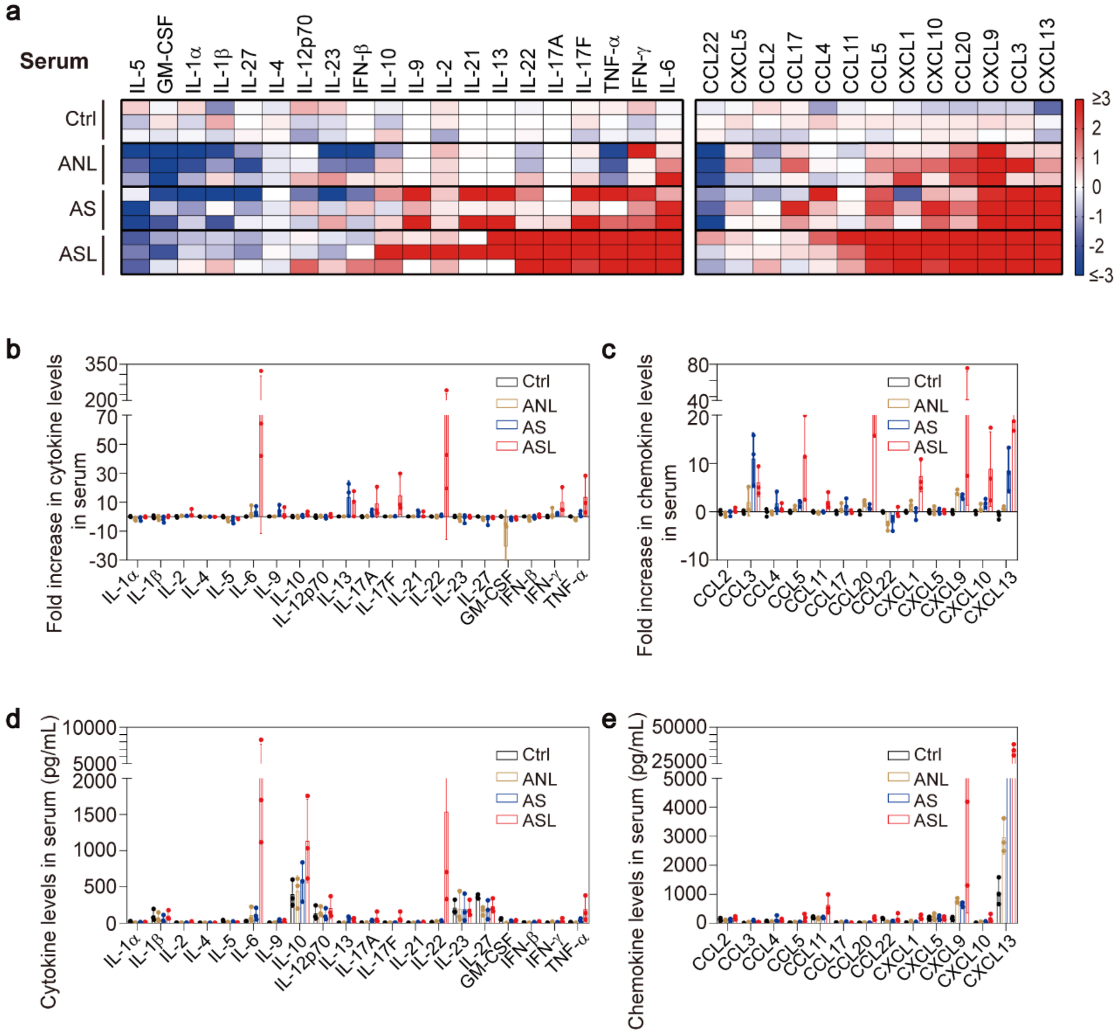

Supplementary Figure 18. Systemic immune responses elicited by Ausome-mediated immune modulation. After two treatments with AS, ANL or ASL, the serum was isolated 6 h after the second laser irradiation and the concentrations of cytokines and chemokines were measured. **a**, Heat map showing the levels of cytokines and chemokines that increased or decreased 3-fold or greater, compared with the control group (n = 3 mice). **b,c**, Folds changes in the upregulated or downregulated cytokines (**b,** n = 3 mice) and chemokines (**c,** n = 3 mice). **d,e**, Serum concentrations of cytokines (**c,** n = 3 mice) and chemokines (**d,** n = 3 mice). The data are presented as the mean ± s.d. Source data are provided as a Source Data file.

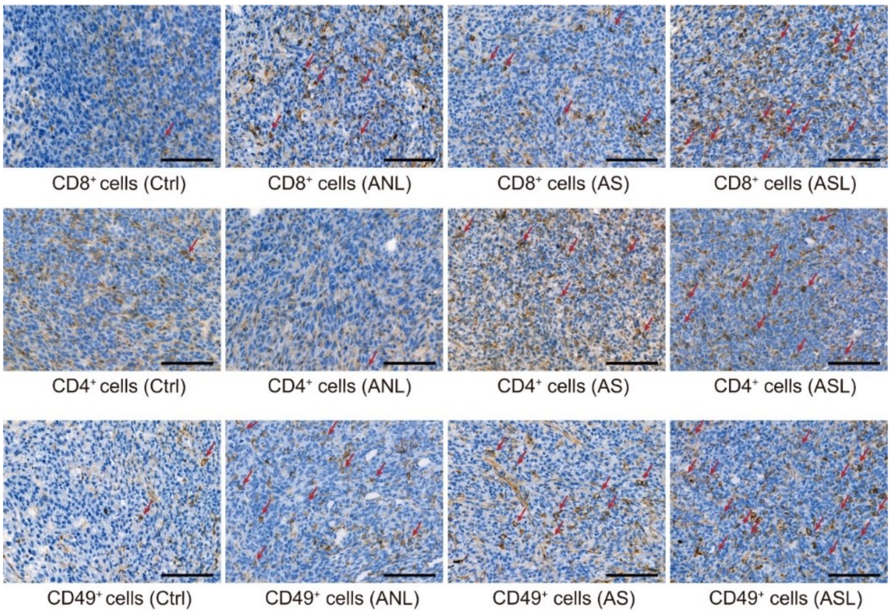

Supplementary Figure 19. Tumor T cell infiltration after Ausome-mediated, multi-layered modulation. Representative immunohistochemical images of tumor sections isolated from mice treated with AS, ANL or ASL, T cells and NK cells with specific antibodies against CD4, CD8 and CD49, respectively. The red arrow indicated positive cells. Scale bar, 100 μm. This experiment was repeated three times independently with similar results.

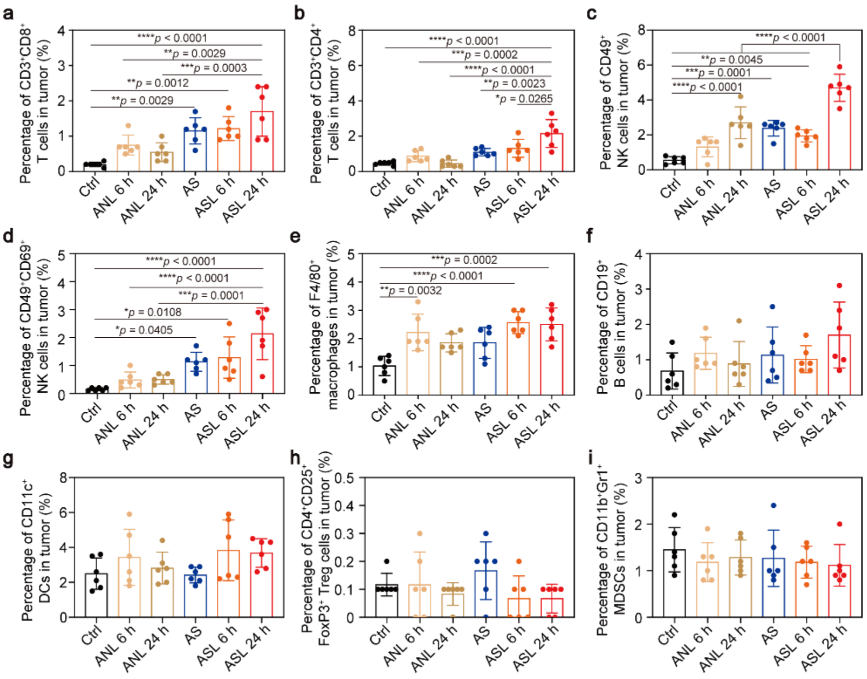

Supplementary Figure 20. Reshaped immune contexture in the tumor microenvironment after Ausome-mediated, multi-layered modulation. **a-i**, Percentage of immune cells in tumor tissues after treatment with AS, ANL or ASL (n = 6 mice). The $CD3^+CD8^+$ T cells (**a**), $CD3^+CD4^+$ T cells (**b**), $CD49^+$ NK cells (**c**), $CD49^+CD69^+$ activated NK cells (**d**), F4/80^+ macrophages (**e**), $CD19^+$ B cells (**f**), $CD11c^+$ DCs (**g**), $CD4^+CD25^+Foxp3^+$ Treg cells (**h**) and $CD11b^+Gr1^+$ MDSCs (**i**) were evaluated at 6 and 24 h after the second laser irradiation by flow cytometry. The data are shown as the mean ± s.d. $*p < 0.05$, $**p < 0.01$, $***p < 0.001$, $****p < 0.0001$; one-way ANOVA followed by the Bonferroni multiple comparison test for statistical significance analysis. Source data are provided as a Source Data file.

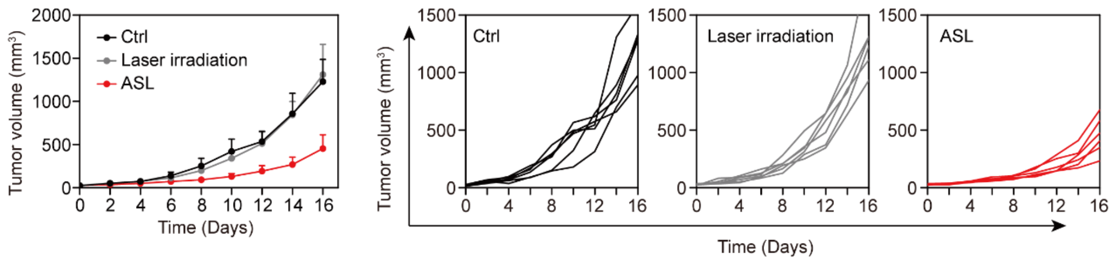

Supplementary Figure 21. Evaluation of the antitumor effects of laser irradiation. Balb/c mice were *in situ* inoculated with 4T1 breast cancer cells. When the volume reached 50-100 mm^3 (day 0), the tumors were exposed to 660 nm laser (1.2 W/cm^2) for 30 min with (ASL group) or without (Laser irradiation group) previously administrated Ausome. Treatments were performed three times, and the average and individual tumor volumes of the 4T1 breast cancer models during the therapeutic experiment (n = 6 mice) are shown. The numerical data are shown as the mean ± s.d. Source data are provided as a Source Data file.

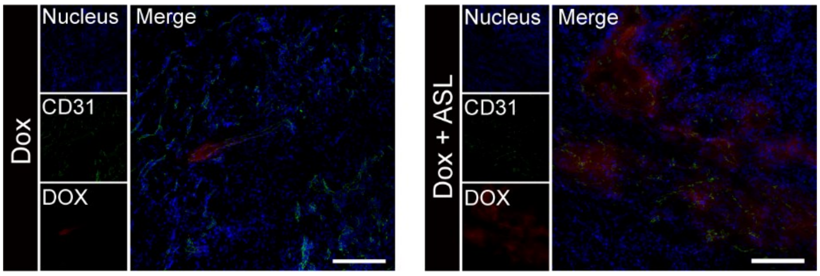

Supplementary Figure 22. Ausome-mediated, hyperthermia-facilitated perfusion of tumor tissue with a chemotherapeutic agent. 4T1 tumor-bearing mice were intravenously injected with Dox (1.5 mg/kg) and treated without or with additional ASL therapy. Tumor sections were immunofluorescence-stained with a specific antibody against CD31 and imaged using a laser scanning confocal microscope (blue: nucleus, green: CD31, red: Dox). Scale bars, 200 μm. This experiment was repeated three times independently with similar results.

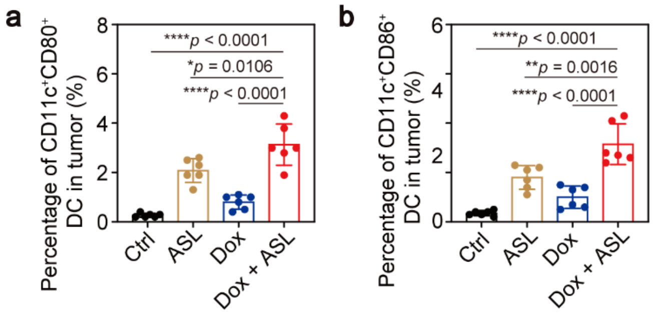

Supplementary Figure 23. Ausome-triggered DC maturation in tumor tissue. **a,b**, Mice were treated with ASL, Dox monotherapy or Dox plus ASL combination therapy, and the intratumoral CD11c$^+$CD80$^+$ (**a,** n = 6 mice) or CD11c$^+$CD86$^+$ (**b,** n = 6 mice) mature DCs were quantified by flow cytometry. The data are shown as the mean ± s.d. $*p < 0.05$, $**p < 0.01$, $***p < 0.001$, $****p < 0.0001$; one-way ANOVA followed by the Bonferroni multiple comparison test were used for statistical significance analysis. Source data are provided as a Source Data file.

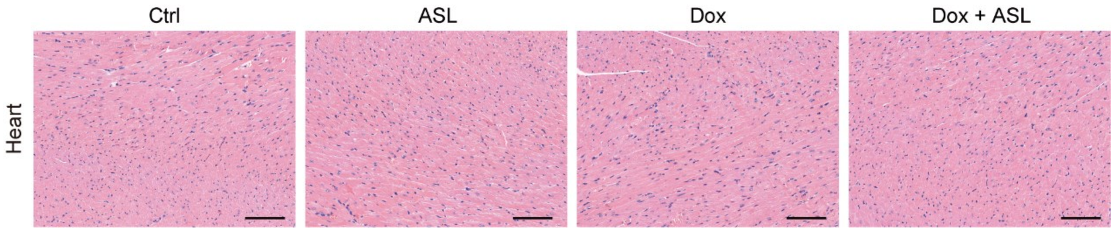

Supplementary Figure 24. Safety evaluation of Dox-including regimens, in which Dox was applied at a low-toxicity dose. Representative H&E stained images of heart sections from mice treated with the procedures presented in **Fig. 5f**. Scale bars, 100 μm. This experiment was repeated three times independently with similar results.

Gating strategy: mature BMDC

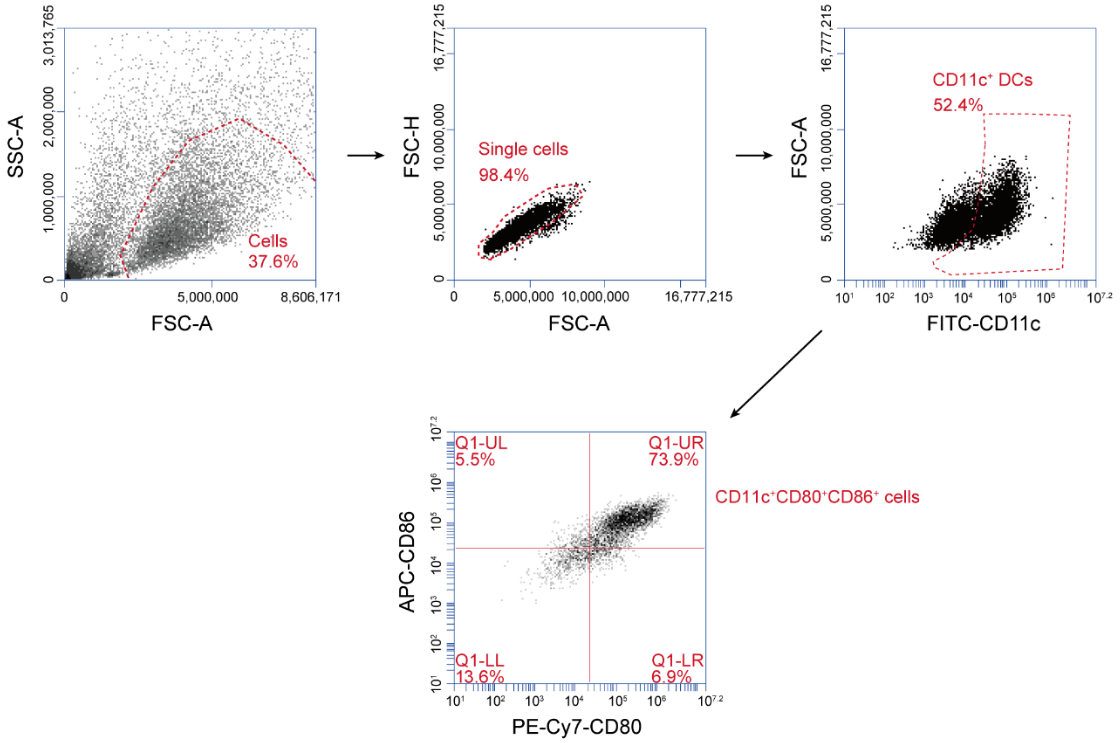

Supplementary Figure 25. Gating strategy for BMDC mature experiments. Related to **Figure 2m, n, Supplementary Figure 9a-d**.

Gating strategy: T cell activation

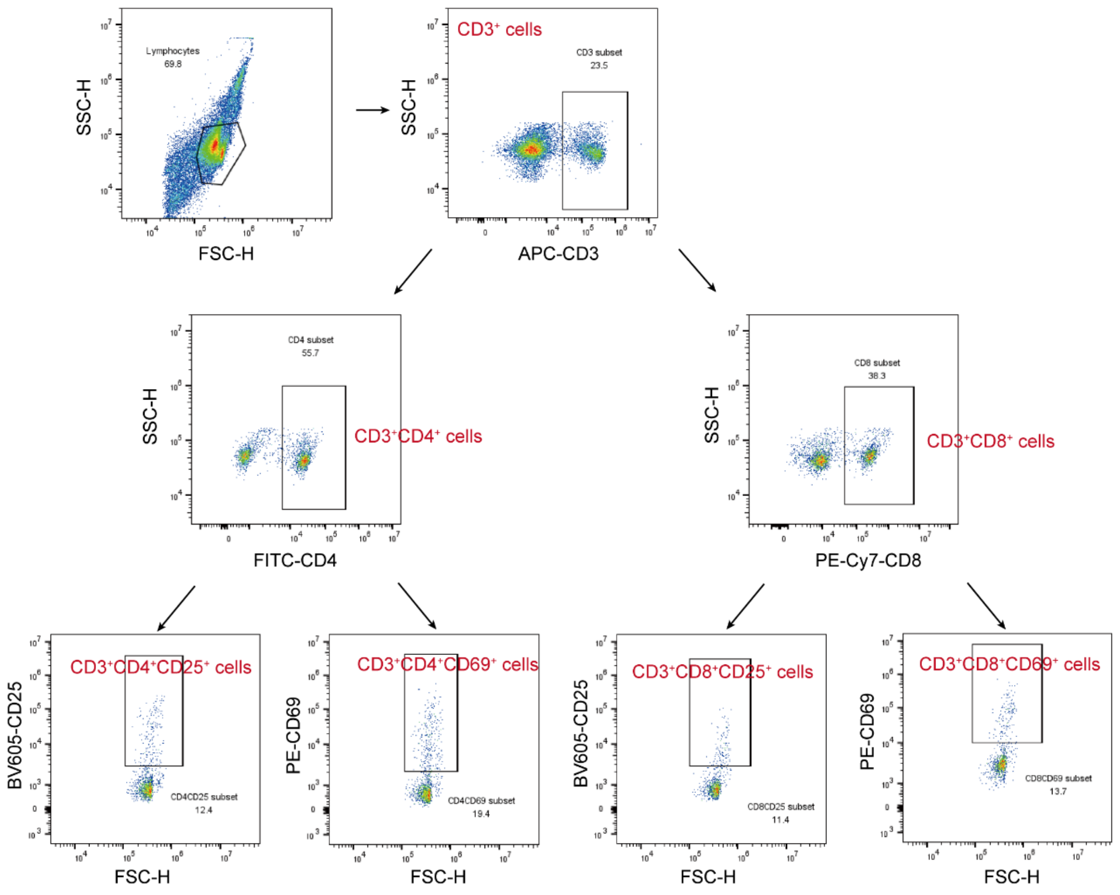

Supplementary Figure 26. Gating strategy for T cell activation. Related to **Figure 2o.**

Gating strategy: Antigen specific T cells and NK cells in blood

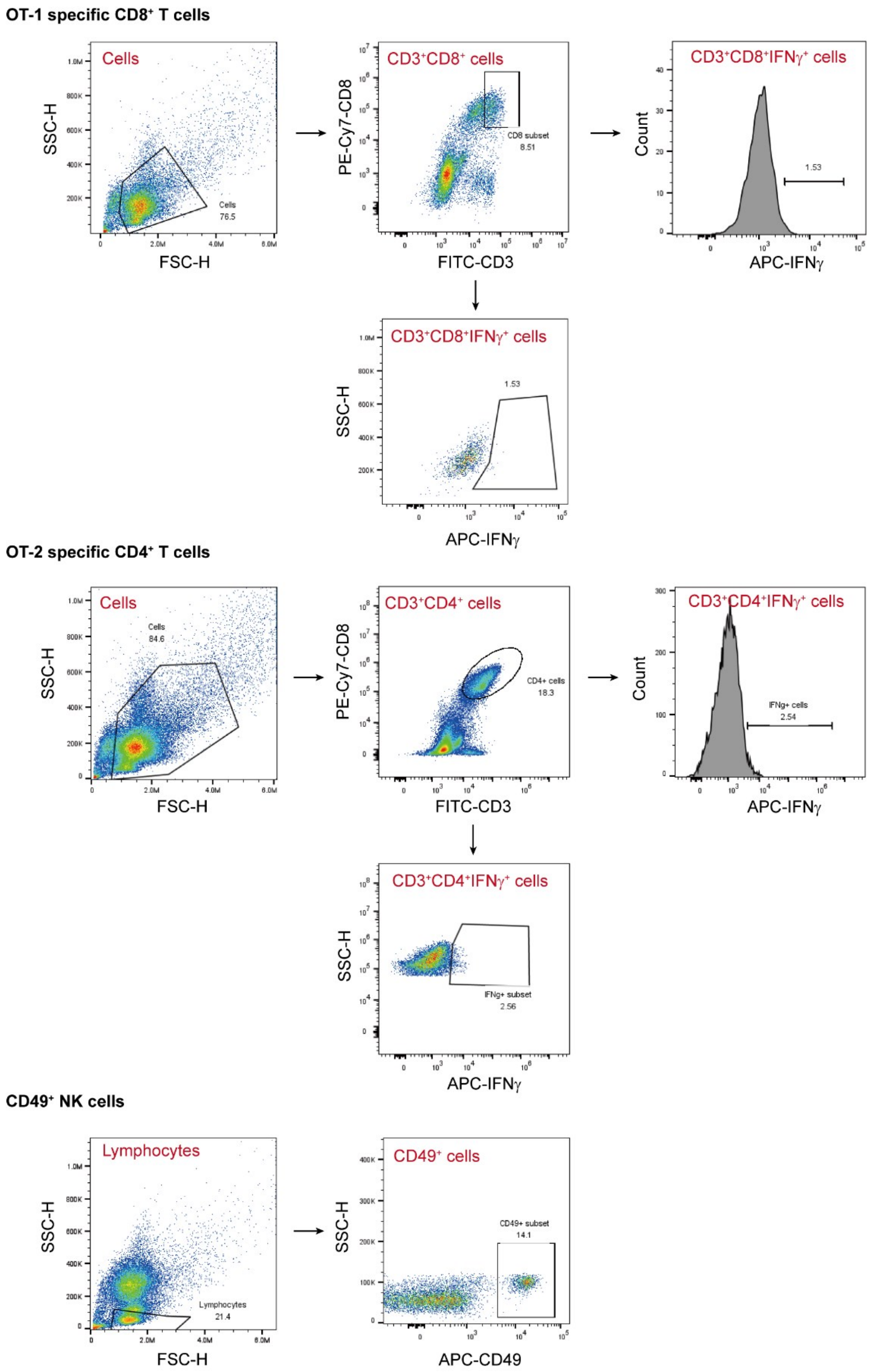

Supplementary Figure 27. Gating strategy for antigen specific T cells and NK cells in blood. Related to **Figure 4e**.

Gating strategy: Tumor infiltrated CFSE labeled OT-1 T cells

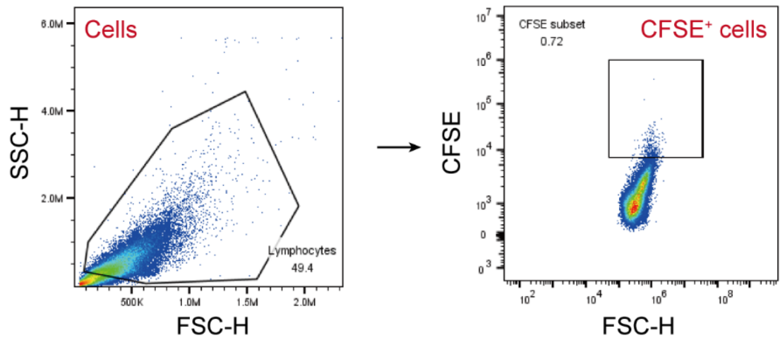

Supplementary Figure 28. Gating strategy for CFSE labeled cells in tumor tissue. Related to **Figure 4g**.

Gating strategy: Tumor infiltrated immune cells

T cells and B cells

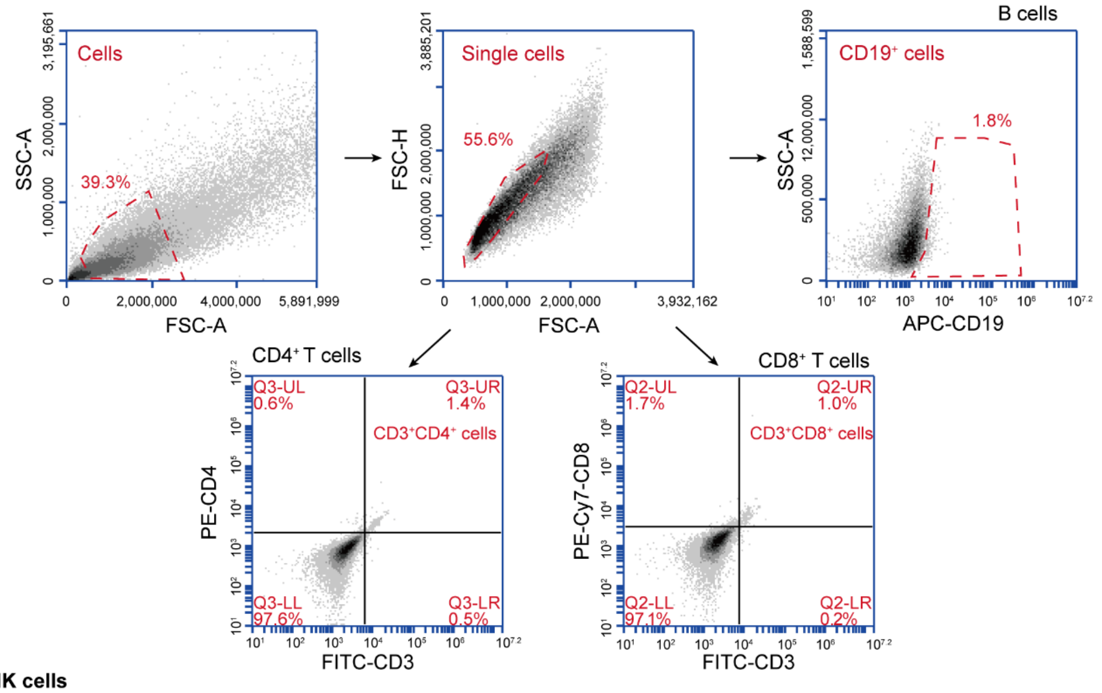

NK cells

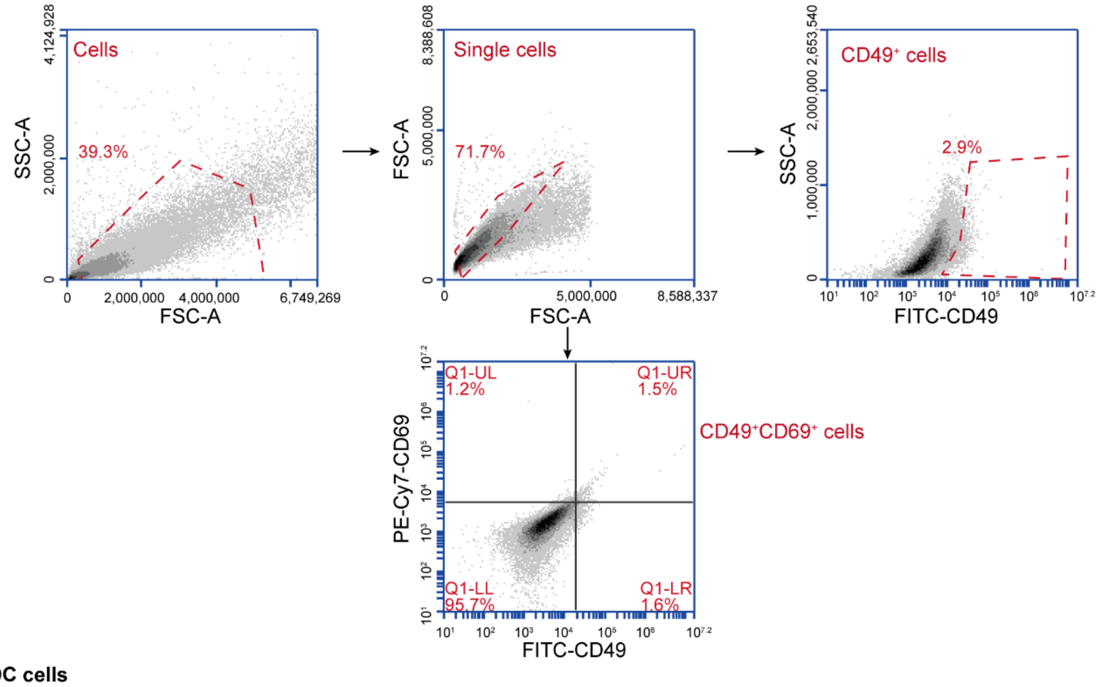

DC cells

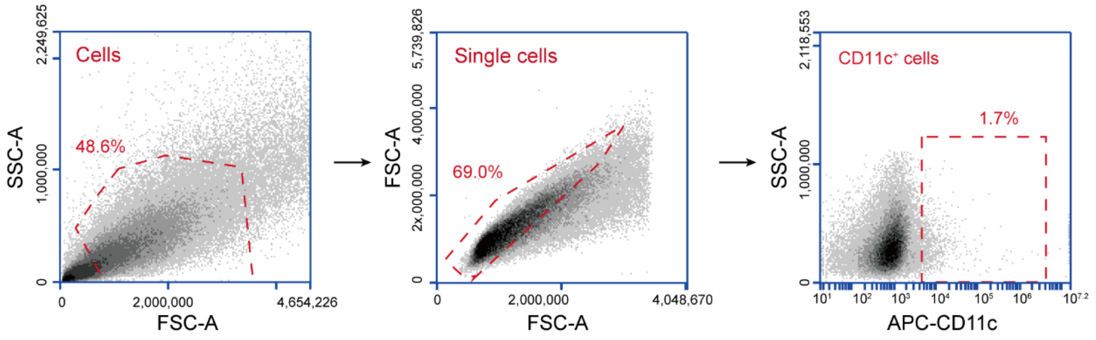

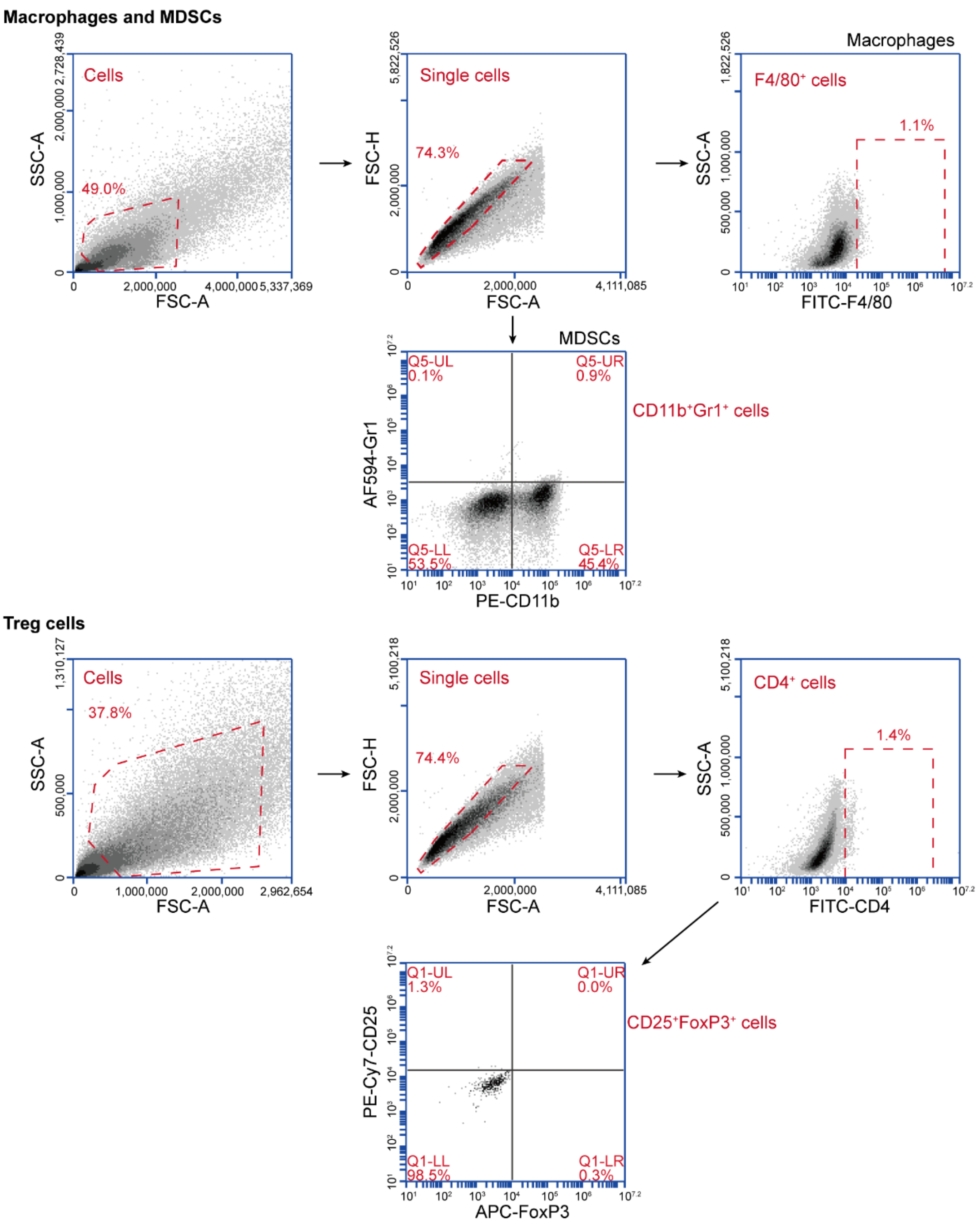

Supplementary Figure 29. Gating strategy for immune cells in tumor tissue. Related to **Figure 4j-l, Figure 5d, i, Supplementary Figure 20**.

Gating strategy: Calreticulin levels on tumor cells

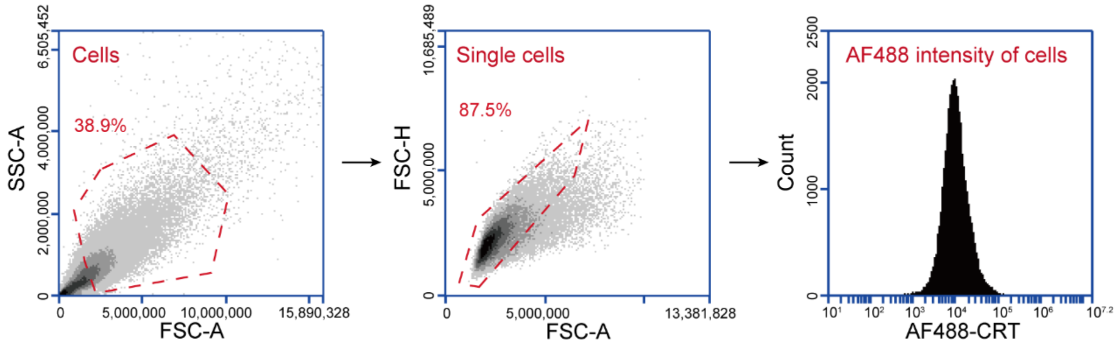

Supplementary Figure 30. Gating strategy for assessing the calreticulin levels on tumor cells. Related to **Figure 5g**.

Gating strategy: Intratumoral mature DC evaluation

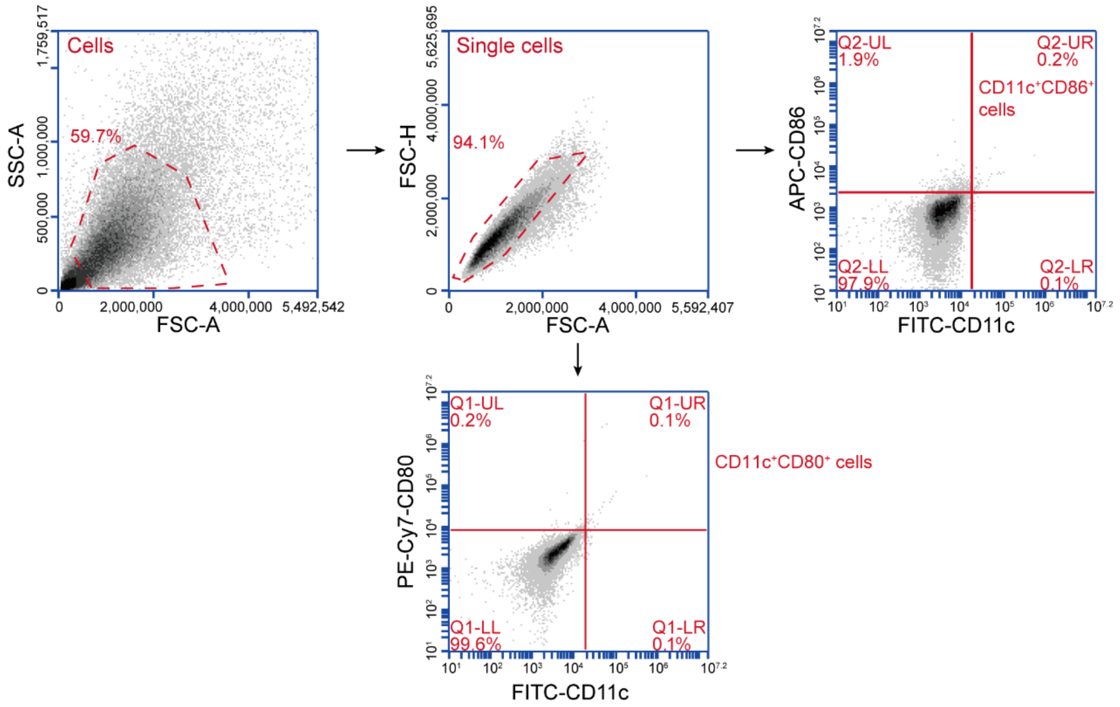

Supplementary Figure 31. Gating strategy for assessing DC mature in tumor tissue. Related to **Supplementary Figure 23**.
