# Supplementary material for: Biosynthesized gold nanoparticles that activate Toll-like receptors and elicit localized light-converting hyperthermia for pleiotropic tumor immunoregulation
Source: Nat Commun. 2023 Aug 24;14:5178. doi: 10.1038/s41467-023-40851-4 (PMC10449932; doi:10.1038/s41467-023-40851-4)
Supplement: Supplementary file 3 — Reporting Summary [file 41467_2023_40851_MOESM3_ESM.pdf]

nature portfolio

Corresponding author(s): Guangjun Nie, Ruifang Zhao

Last updated by author(s): Jun 15, 2023

Reporting Summary

Nature Portfolio wishes to improve the reproducibility of the work that we publish. This form provides structure for consistency and transparency in reporting. For further information on Nature Portfolio policies, see our Editorial Policies and the Editorial Policy Checklist.

Statistics

For all statistical analyses, confirm that the following items are present in the figure legend, table legend, main text, or Methods section.

n/a	Confirmed	
☐	☒	The exact sample size (n) for each experimental group/condition, given as a discrete number and unit of measurement
☐	☒	A statement on whether measurements were taken from distinct samples or whether the same sample was measured repeatedly
☐	☒	The statistical test(s) used AND whether they are one- or two-sided<br>*Only common tests should be described solely by name; describe more complex techniques in the Methods section.*
☒	☐	A description of all covariates tested
☐	☒	A description of any assumptions or corrections, such as tests of normality and adjustment for multiple comparisons
☐	☒	A full description of the statistical parameters including central tendency (e.g. means) or other basic estimates (e.g. regression coefficient) AND variation (e.g. standard deviation) or associated estimates of uncertainty (e.g. confidence intervals)
☐	☒	For null hypothesis testing, the test statistic (e.g. F, t, r) with confidence intervals, effect sizes, degrees of freedom and P value noted<br>*Give P values as exact values whenever suitable.*
☒	☐	For Bayesian analysis, information on the choice of priors and Markov chain Monte Carlo settings
☒	☐	For hierarchical and complex designs, identification of the appropriate level for tests and full reporting of outcomes
☒	☐	Estimates of effect sizes (e.g. Cohen's d, Pearson's r), indicating how they were calculated

Our web collection on statistics for biologists contains articles on many of the points above.

Software and code

Policy information about availability of computer code

Data collection	Zetasizer Software (version 8. 01. 4906) was used to collect data of size and zeta potential of nanoparticles; BD Accuri C6 Software (version 1.0.264.21) and Attune NxT Flow Cytometer Software (version 2.6) were used to collect data of flow cytometry; Living Image software (version 4.3.1) was used for imaging and quantifying the fluorescence in mice; SmartView Software (version 4.3.122.0) was used to monitor the temperature in photothermal related experiments.
Data analysis	BD Accuri C6 Software (version 1.0.264.21) and FlowJo (version 10.8.1) was used to analyze the data of flow cytometry; GraphPad Prism (version 8.4.2) was used for the statistical analysis.

For manuscripts utilizing custom algorithms or software that are central to the research but not yet described in published literature, software must be made available to editors and reviewers. We strongly encourage code deposition in a community repository (e.g. GitHub). See the Nature Portfolio guidelines for submitting code & software for further information.

Data

Policy information about availability of data

All manuscripts must include a data availability statement. This statement should provide the following information, where applicable:

- Accession codes, unique identifiers, or web links for publicly available datasets
- A description of any restrictions on data availability
- For clinical datasets or third party data, please ensure that the statement adheres to our policy

All data supporting the results are provided within the manuscript, the Supplementary information files or the Source data file. The RNA-Seq data has been

deposited in the NCBI database under accession code PRJNA978837. The mass spectrometry proteomics data have been deposited to the ProteomeXchange Consortium via the PRIDE [1] partner repository with the dataset identifier PXD044359. Source data are provided as additional file with this paper.

Research involving human participants, their data, or biological material

Policy information about studies with human participants or human data. See also policy information about sex, gender (identity/presentation), and sexual orientation and race, ethnicity and racism.

Reporting on sex and gender	This study has no human participants.
Reporting on race, ethnicity, or other socially relevant groupings	This study has no human participants.
Population characteristics	This study has no human participants.
Recruitment	This study has no human participants.
Ethics oversight	This study has no human participants.

Note that full information on the approval of the study protocol must also be provided in the manuscript.

Field-specific reporting

Please select the one below that is the best fit for your research. If you are not sure, read the appropriate sections before making your selection.

☒ Life sciences ☐ Behavioural & social sciences ☐ Ecological, evolutionary & environmental sciences

For a reference copy of the document with all sections, see nature.com/documents/nr-reporting-summary-flat.pdf

Life sciences study design

All studies must disclose on these points even when the disclosure is negative.

Sample size	No statistical method was used to predetermine sample size in this study. The sample size was determined according to the previous research in this field (references: Xu. et al. Nature Nanotechnology. 2020. 15:1043-1052; Cheng. et al. Nature communications. 2021. 2041; Kuai. et al. Nature Materials. 2017. 16(4):489-496; Qing. et al. Advanced Materials. 2020. 2002085;). The in vitro experiments were performed with at least three biological independent samples and the in vivo experiments were performed with 3-6 animals, which were sufficient for statistic analysis and minimizing the using of animals/reagents.
Data exclusions	No data was excluded.
Replication	All data came from at least three biological independent replications and presented as mean ± SD. All attempts at replication were successful.
Randomization	All animals were numbered and randomly allocated into each group by random number table method. For cell line based experiments, randomization was not applied due to all samples were performed equally.
Blinding	All investigators were blinded to group allocation during data collection and analysis.

Behavioural & social sciences study design

All studies must disclose on these points even when the disclosure is negative.

Study description	*Briefly describe the study type including whether data are quantitative, qualitative, or mixed-methods (e.g. qualitative cross-sectional, quantitative experimental, mixed-methods case study).*
Research sample	*State the research sample (e.g. Harvard university undergraduates, villagers in rural India) and provide relevant demographic information (e.g. age, sex) and indicate whether the sample is representative. Provide a rationale for the study sample chosen. For studies involving existing datasets, please describe the dataset and source.*
Sampling strategy	*Describe the sampling procedure (e.g. random, snowball, stratified, convenience). Describe the statistical methods that were used to predetermine sample size OR if no sample-size calculation was performed, describe how sample sizes were chosen and provide a rationale for why these sample sizes are sufficient. For qualitative data, please indicate whether data saturation was considered, and what criteria were used to decide that no further sampling was needed.*
Data collection	*Provide details about the data collection procedure, including the instruments or devices used to record the data (e.g. pen and paper, computer, eye tracker, video or audio equipment) whether anyone was present besides the participant(s) and the researcher, and whether the researcher was blind to experimental condition and/or the study hypothesis during data collection.*

Timing	*Indicate the start and stop dates of data collection. If there is a gap between collection periods, state the dates for each sample cohort.*
Data exclusions	*If no data were excluded from the analyses, state so OR if data were excluded, provide the exact number of exclusions and the rationale behind them, indicating whether exclusion criteria were pre-established.*
Non-participation	*State how many participants dropped out/declined participation and the reason(s) given OR provide response rate OR state that no participants dropped out/declined participation.*
Randomization	*If participants were not allocated into experimental groups, state so OR describe how participants were allocated to groups, and if allocation was not random, describe how covariates were controlled.*

Ecological, evolutionary & environmental sciences study design

All studies must disclose on these points even when the disclosure is negative.

Study description	*Briefly describe the study. For quantitative data include treatment factors and interactions, design structure (e.g. factorial, nested, hierarchical), nature and number of experimental units and replicates.*
Research sample	*Describe the research sample (e.g. a group of tagged Passer domesticus, all Stenocereus thurberi within Organ Pipe Cactus National Monument), and provide a rationale for the sample choice. When relevant, describe the organism taxa, source, sex, age range and any manipulations. State what population the sample is meant to represent when applicable. For studies involving existing datasets, describe the data and its source.*
Sampling strategy	*Note the sampling procedure. Describe the statistical methods that were used to predetermine sample size OR if no sample-size calculation was performed, describe how sample sizes were chosen and provide a rationale for why these sample sizes are sufficient.*
Data collection	*Describe the data collection procedure, including who recorded the data and how.*
Timing and spatial scale	*Indicate the start and stop dates of data collection, noting the frequency and periodicity of sampling and providing a rationale for these choices. If there is a gap between collection periods, state the dates for each sample cohort. Specify the spatial scale from which the data are taken*
Data exclusions	*If no data were excluded from the analyses, state so OR if data were excluded, describe the exclusions and the rationale behind them, indicating whether exclusion criteria were pre-established.*
Reproducibility	*Describe the measures taken to verify the reproducibility of experimental findings. For each experiment, note whether any attempts to repeat the experiment failed OR state that all attempts to repeat the experiment were successful.*
Randomization	*Describe how samples/organisms/participants were allocated into groups. If allocation was not random, describe how covariates were controlled. If this is not relevant to your study, explain why.*
Blinding	*Describe the extent of blinding used during data acquisition and analysis. If blinding was not possible, describe why OR explain why blinding was not relevant to your study.*

Did the study involve field work? ☐ Yes ☐ No

Field work, collection and transport

Field conditions	*Describe the study conditions for field work, providing relevant parameters (e.g. temperature, rainfall).*
Location	*State the location of the sampling or experiment, providing relevant parameters (e.g. latitude and longitude, elevation, water depth).*
Access & import/export	*Describe the efforts you have made to access habitats and to collect and import/export your samples in a responsible manner and in compliance with local, national and international laws, noting any permits that were obtained (give the name of the issuing authority, the date of issue, and any identifying information).*
Disturbance	*Describe any disturbance caused by the study and how it was minimized.*

Reporting for specific materials, systems and methods

We require information from authors about some types of materials, experimental systems and methods used in many studies. Here, indicate whether each material, system or method listed is relevant to your study. If you are not sure if a list item applies to your research, read the appropriate section before selecting a response.

Materials & experimental systems

n/a	Involved in the study
☐	☒ Antibodies
☐	☒ Eukaryotic cell lines
☒	☐ Palaeontology and archaeology
☐	☒ Animals and other organisms
☒	☐ Clinical data
☒	☐ Dual use research of concern
☒	☐ Plants

Methods

n/a	Involved in the study
☒	☐ ChIP-seq
☐	☒ Flow cytometry
☒	☐ MRI-based neuroimaging

Antibodies

Antibodies used

The FITC-anti-mouse CD11c (catalogue No: 117305, clone name: N418 1:100), PE-Cy7-anti-mouse CD80 (catalogue No: 104734, clone name: 16-10A1 1:100), APC-anti-mouse CD86 (catalogue No: 105116, clone name: PO3 1:100), FITC-anti-mouse CD3 (catalogue No: 100203, clone name: 17A2 1:100), PE-anti-mouse CD4 (catalogue No: 100407, clone name: GK1.5 1:100), PE-Cy7-anti-mouse CD8 (catalogue No: 100722, clone name: 53-6.7 1:100), PE-anti-mouse-CD69 (catalogue No: 104508, clone name: H1.2F3 1:100), Brilliant Violet 605-anti-mouse-CD25 (catalogue No: 102035, clone name: PC61 1:100), APC-anti-mouse CD19 (catalogue No: 152410, clone name: 1D3/CD19 1:100), FITC-anti-mouse CD49 (catalogue No: 108905, clone name: DX5 1:100), PE-Cy7-anti-mouse-CD69 (catalogue No: 104512, clone name: H1.2F3 1:100), APC-anti-mouse-CD11c (catalogue No: 117310, clone name: N418 1:100), FITC-anti-mouse CD4 (catalogue No: 100405, clone name: GK1.5 1:100), PE-Cy7-anti-mouse CD25 (catalogue No: 101916, clone name: 3C7 1:100), Alexa Fluor 647-anti-mouse Foxp3 (catalogue No: 126408, clone name: MF-14 1:100), FITC-anti-mouse F4/80 (catalogue No: 123108, clone name: BM8 1:100), PE-anti-mouse-CD11b (catalogue No: 101208, clone name: M1-70 1:100), Alexa Fluor 594-anti-mouse Gr1 (catalogue No: 108448, clone name: RB6-8C5 1:100) were purchased from Biolegend (San Diego, USA).

The anti-mouse Hsp 70 (catalogue No: ab2787, clone name: 5A5, 1:100), anti-mouse Hsp 90 (catalogue No: ab32568, clone name: E296, 1:100), anti-mouse ICAM-1 (catalogue No: ab222736, clone name: EPR22161-284, 1:1000), anti-mouse VACM-1 (catalogue No: ab134047, clone name: EPR5047, 1:5000) were purchased from Abcam (Cambridge, UK).

Anti-mouse CD49 (catalogue No: 142602, clone name: HMα1 1:100), anti-mouse CD31 (catalogue No: 102402, clone name: 390 1:100) were purchased from Biolegend (San Diego, USA).

Anti-mouse PD-L1 (catalogue No: 28076-1-AP, 1:500) were purchased from Proteintech (Manchester, UK).

Alexa Fluor 488-anti-mouse calreticulin (catalogue No: 142602, 1:100) was obtained from Cell Signaling Technology (CST, Massachusetts, USA).

The InVivoPlus anti-mouse PD-1 blocking antibody (catalogue No: BP0146, clone name: RMP1-14 7.5mg/kg for in vivo use) was purchased from BioXCell (Lebanon, USA).

Validation

https://www.biolegend.com/en-us/products/fitc-anti-mouse-cd11c-antibody-1815
https://www.biolegend.com/en-us/products/pe-cyanine7-anti-mouse-cd80-antibody-9320
https://www.biolegend.com/en-us/products/pe-cyanine7-anti-mouse-cd86-antibody-3045
https://www.biolegend.com/en-us/products/fitc-anti-mouse-cd3-antibody-45
https://www.biolegend.com/en-us/products/pe-anti-mouse-cd4-antibody-250
https://www.biolegend.com/en-us/products/pe-cyanine7-anti-mouse-cd8a-antibody-1906
https://www.biolegend.com/en-us/products/pe-anti-mouse-cd69-antibody-265
https://www.biolegend.com/en-us/products/brilliant-violet-605-anti-mouse-cd25-antibody-7639
https://www.biolegend.com/en-us/products/apc-anti-mouse-cd19-antibody-13680
https://www.biolegend.com/en-us/products/fitc-anti-mouse-cd49b-pan-nk-cells-antibody-233
https://www.biolegend.com/en-us/products/pe-cyanine7-anti-mouse-cd69-antibody-3168
https://www.biolegend.com/en-us/products/apc-anti-mouse-cd11c-antibody-1813
https://www.biolegend.com/en-us/products/fitc-anti-mouse-cd4-antibody-248
https://www.biolegend.com/en-us/products/pe-cyanine7-anti-mouse-cd25-antibody-13235
https://www.biolegend.com/en-us/products/alexa-fluor-647-anti-mouse-foxp3-antibody-4662
https://www.biolegend.com/en-us/products/fitc-anti-mouse-f4-80-antibody-4067
https://www.biolegend.com/en-us/products/pe-anti-mouse-human-cd11b-antibody-349
https://www.biolegend.com/en-us/products/alexa-fluor-594-anti-mouse-ly-6g-ly-6c-gr-1-antibody-9672

https://www.abcam.cn/products/primary-antibodies/hsp70-antibody-5a5-ab2787.html
https://www.abcam.cn/products/primary-antibodies/hsp90-beta-antibody-e296-ab32568.html
https://www.abcam.cn/products/primary-antibodies/icam1-antibody-epr22161-284-ab222736.html
https://www.abcam.cn/products/primary-antibodies/vcam1-antibody-epr5047-ab134047.html

https://www.biolegend.com/en-us/products/purified-anti-mouse-cd49a-antibody-7534

https://www.biolegend.com/en-us/products/purified-anti-mouse-cd31-antibody-123

https://www.ptgcn.com/products/PD-L1-CD274-Antibody-28076-1-AP.htm#product-information

https://www.cellsignal.cn/products/antibody-conjugates/calreticulin-d3e6-xp-rabbit-mab-alexa-fluor-488-conjugate/62304?site-search type=Products&N=4294956287&Ntt=62304s&fromPage=plp&_requestid=3093218

https://bioxcell.com/invivoplus-anti-mouse-pd-1-cd279-bp0146

Eukaryotic cell lines

Policy information about cell lines and Sex and Gender in Research

Cell line source(s)	4T1 mouse mammary cancer cells, mouse colon carcinoma CT26 cells and human umbilical vein endothelial cells (HUVEC) were originally purchased from the American Type Culture Collection (ATCC, MA, USA). The 4T1-OVA cells was constructed from 4T1 cells with stable transfection of ovalbumin and originally obtained from Keruisibo (Beijing, China).
Authentication	The cell lines were authenticated by the manufacturers and supported by multiple publications. In this study, we did not perform authentication on these cells.
Mycoplasma contamination	The cells were regularly performed with mycoplasma detection and were confirmed negative for mycoplasma contamination.
Commonly misidentified lines (See ICLAC register)	No commonly misidentified cell lines were used in this study.

Palaeontology and Archaeology

Specimen provenance	*Provide provenance information for specimens and describe permits that were obtained for the work (including the name of the issuing authority, the date of issue, and any identifying information). Permits should encompass collection and, where applicable, export.*
Specimen deposition	*Indicate where the specimens have been deposited to permit free access by other researchers.*
Dating methods	*If new dates are provided, describe how they were obtained (e.g. collection, storage, sample pretreatment and measurement), where they were obtained (i.e. lab name), the calibration program and the protocol for quality assurance OR state that no new dates are provided.*

☐ Tick this box to confirm that the raw and calibrated dates are available in the paper or in Supplementary Information.

Ethics oversight	*Identify the organization(s) that approved or provided guidance on the study protocol, OR state that no ethical approval or guidance was required and explain why not.*

Note that full information on the approval of the study protocol must also be provided in the manuscript.

Animals and other research organisms

Policy information about studies involving animals; ARRIVE guidelines recommended for reporting animal research, and Sex and Gender in Research

Laboratory animals	6-8 weeks-old female Balb/c mice, 6-8 weeks-old female and male Sprague Dawley rats, were purchased from Vital River Experimental Animal Technology Co. Ltd (Beijing, China), 6-8 weeks-old female Jackson Laboratories sourced transgenic mice [C57BL/6-Tg (TcraTcrb)1100Mjb/J] with MHC Ⅰ restricted and OVA257-264 specific TCR populations were purchased from Aniphe Biolaboratory Inc. (Jiangsu, China).
Wild animals	No wild animals involved in this study.
Reporting on sex	The current study emphasized the photothermal effects, immunoregulating abilities and anti-tumor outcomes of Ausome, which should not be significantly affected by sex. We used female mice mostly in this study for generating breast cancer model, which is easy to develop in situ and suitable to verify the effects of laser dependent photothermal therapy. Nevertheless, we supplemented safety assessments on both female and male rats, no obvious differences were observed. Therefore, sex is not a consideration in this study and these results could be applied to both sexes.
Field-collected samples	No field-collected samples involved in this study.
Ethics oversight	All animal-involving procedures were performed in compliance with experimental guidelines approved by the Animal Care and Welfare Committee of the National Centre for Nanoscience and Technology.

Note that full information on the approval of the study protocol must also be provided in the manuscript.

Clinical data

Policy information about clinical studies

All manuscripts should comply with the ICMJE guidelines for publication of clinical research and a completed CONSORT checklist must be included with all submissions.

Clinical trial registration — *Provide the trial registration number from ClinicalTrials.gov or an equivalent agency.*

Study protocol — *Note where the full trial protocol can be accessed OR if not available, explain why.*

Data collection — *Describe the settings and locales of data collection, noting the time periods of recruitment and data collection.*

Outcomes — *Describe how you pre-defined primary and secondary outcome measures and how you assessed these measures.*

Dual use research of concern

Policy information about dual use research of concern

Hazards

Could the accidental, deliberate or reckless misuse of agents or technologies generated in the work, or the application of information presented in the manuscript, pose a threat to:

No	Yes	
☐	☐	Public health
☐	☐	National security
☐	☐	Crops and/or livestock
☐	☐	Ecosystems
☐	☐	Any other significant area

Experiments of concern

Does the work involve any of these experiments of concern:

No	Yes	
☐	☐	Demonstrate how to render a vaccine ineffective
☐	☐	Confer resistance to therapeutically useful antibiotics or antiviral agents
☐	☐	Enhance the virulence of a pathogen or render a nonpathogen virulent
☐	☐	Increase transmissibility of a pathogen
☐	☐	Alter the host range of a pathogen
☐	☐	Enable evasion of diagnostic/detection modalities
☐	☐	Enable the weaponization of a biological agent or toxin
☐	☐	Any other potentially harmful combination of experiments and agents

Plants

Seed stocks — *Report on the source of all seed stocks or other plant material used. If applicable, state the seed stock centre and catalogue number. If plant specimens were collected from the field, describe the collection location, date and sampling procedures.*

Novel plant genotypes — *Describe the methods by which all novel plant genotypes were produced. This includes those generated by transgenic approaches, gene editing, chemical/radiation-based mutagenesis and hybridization. For transgenic lines, describe the transformation method, the number of independent lines analyzed and the generation upon which experiments were performed. For gene-edited lines, describe the editor used, the endogenous sequence targeted for editing, the targeting guide RNA sequence (if applicable) and how the editor was applied.*

Authentication — *Describe any authentication procedures for each seed stock used or novel genotype generated. Describe any experiments used to assess the effect of a mutation and, where applicable, how potential secondary effects (e.g. second site T-DNA insertions, mosiacism, off-target gene editing) were examined.*

ChIP-seq

Data deposition

☐ Confirm that both raw and final processed data have been deposited in a public database such as GEO.

☐ Confirm that you have deposited or provided access to graph files (e.g. BED files) for the called peaks.

Data access links<br>*May remain private before publication.*	*For "Initial submission" or "Revised version" documents, provide reviewer access links. For your "Final submission" document, provide a link to the deposited data.*
Files in database submission	*Provide a list of all files available in the database submission.*
Genome browser session<br>(e.g. UCSC)	*Provide a link to an anonymized genome browser session for "Initial submission" and "Revised version" documents only, to enable peer review. Write "no longer applicable" for "Final submission" documents.*

Methodology

Replicates	*Describe the experimental replicates, specifying number, type and replicate agreement.*
Sequencing depth	*Describe the sequencing depth for each experiment, providing the total number of reads, uniquely mapped reads, length of reads and whether they were paired- or single-end.*
Antibodies	*Describe the antibodies used for the ChIP-seq experiments; as applicable, provide supplier name, catalog number, clone name, and lot number.*
Peak calling parameters	*Specify the command line program and parameters used for read mapping and peak calling, including the ChIP, control and index files used.*
Data quality	*Describe the methods used to ensure data quality in full detail, including how many peaks are at FDR 5% and above 5-fold enrichment.*
Software	*Describe the software used to collect and analyze the ChIP-seq data. For custom code that has been deposited into a community repository, provide accession details.*

Flow Cytometry

Plots

Confirm that:

☒ The axis labels state the marker and fluorochrome used (e.g. CD4-FITC).

☒ The axis scales are clearly visible. Include numbers along axes only for bottom left plot of group (a 'group' is an analysis of identical markers).

☒ All plots are contour plots with outliers or pseudocolor plots.

☒ A numerical value for number of cells or percentage (with statistics) is provided.

Methodology

Sample preparation	For tumor samples, tissues were isolated and mechanically dissociated into small pieces, followed by digesting in 1640 medium supplemented with 2% FBS, 1mg/mL collagenase IV and 0.1 mg/mL DNase. After 30 min digestion, tissues were pushed through a 70 µm cell strainer to generate single cell suspensions, which were then collected using centrifugation and stained with fluorescence labeled antibodies for 20 min at 4°C. After washing with FACS buffer (1640 medium with 2% FBS), the cells were analyzed using a flow cytometry.<br>For intracellular protein staining, single cells were firstly fixed in fixation solution for 20 min at 4°C, then cells were perforated using a permeabilization buffer. Fluorescence labeled antibodies were diluted in permeabilization buffer and incubated with cells for 20 min at 4°C. The cells were then washed using FACS buffer and analyzed by flow cytometry.
Instrument	BD Accuri C6 flow cytometer, Beckman CytoFLEX LX Flow Cytometer
Software	BD Accuri C6 Software (version 1.0.264.21), FlowJo (version 10.8.1)
Cell population abundance	At least 10,000 cells were collected when analyzing cell samples; At least 100,000 cells were gated when analyzing tissue samples.
Gating strategy	The gating strategies used in this study haves been provided in supplementary files. The positive and negative populations are defined according to the expression levels (fluorescent intensity) of markers in the analysis.

☒ Tick this box to confirm that a figure exemplifying the gating strategy is provided in the Supplementary Information.

Magnetic resonance imaging

Experimental design

Design type	*Indicate task or resting state; event-related or block design.*
Design specifications	*Specify the number of blocks, trials or experimental units per session and/or subject, and specify the length of each trial or block (if trials are blocked) and interval between trials.*
Behavioral performance measures	*State number and/or type of variables recorded (e.g. correct button press, response time) and what statistics were used to establish that the subjects were performing the task as expected (e.g. mean, range, and/or standard deviation across subjects).*

Acquisition

Imaging type(s)	*Specify: functional, structural, diffusion, perfusion.*
Field strength	*Specify in Tesla*
Sequence & imaging parameters	*Specify the pulse sequence type (gradient echo, spin echo, etc.), imaging type (EPI, spiral, etc.), field of view, matrix size, slice thickness, orientation and TE/TR/flip angle.*
Area of acquisition	*State whether a whole brain scan was used OR define the area of acquisition, describing how the region was determined.*

Diffusion MRI ☐ Used ☐ Not used

Preprocessing

Preprocessing software	*Provide detail on software version and revision number and on specific parameters (model/functions, brain extraction, segmentation, smoothing kernel size, etc.).*
Normalization	*If data were normalized/standardized, describe the approach(es): specify linear or non-linear and define image types used for transformation OR indicate that data were not normalized and explain rationale for lack of normalization.*
Normalization template	*Describe the template used for normalization/transformation, specifying subject space or group standardized space (e.g. original Talairach, MNI305, ICBM152) OR indicate that the data were not normalized.*
Noise and artifact removal	*Describe your procedure(s) for artifact and structured noise removal, specifying motion parameters, tissue signals and physiological signals (heart rate, respiration).*
Volume censoring	*Define your software and/or method and criteria for volume censoring, and state the extent of such censoring.*

Statistical modeling & inference

Model type and settings	*Specify type (mass univariate, multivariate, RSA, predictive, etc.) and describe essential details of the model at the first and second levels (e.g. fixed, random or mixed effects; drift or auto-correlation).*
Effect(s) tested	*Define precise effect in terms of the task or stimulus conditions instead of psychological concepts and indicate whether ANOVA or factorial designs were used.*

Specify type of analysis: ☐ Whole brain ☐ ROI-based ☐ Both

Statistic type for inference (See Eklund et al. 2016)	*Specify voxel-wise or cluster-wise and report all relevant parameters for cluster-wise methods.*
Correction	*Describe the type of correction and how it is obtained for multiple comparisons (e.g. FWE, FDR, permutation or Monte Carlo).*

Models & analysis

n/a	Involved in the study
☐	☐ Functional and/or effective connectivity
☐	☐ Graph analysis
☐	☐ Multivariate modeling or predictive analysis

Functional and/or effective connectivity	*Report the measures of dependence used and the model details (e.g. Pearson correlation, partial correlation, mutual information).*
Graph analysis	*Report the dependent variable and connectivity measure, specifying weighted graph or binarized graph, subject- or group-level, and the global and/or node summaries used (e.g. clustering coefficient, efficiency, etc.).*
Multivariate modeling and predictive analysis	*Specify independent variables, features extraction and dimension reduction, model, training and evaluation metrics.*
